# A new insight into the impact of copy number variations on cell cycle deregulation of luminal-type breast cancer

**DOI:** 10.3389/or.2025.1516409

**Published:** 2025-02-12

**Authors:** Amir Mahdi Khamaneh, Davoud Jafari-Gharabaghlou, Khalil Ansarin, Pouya Pazooki, Zahra Akbarpour, Behrooz Naghili, Nosratollah Zarghami

**Affiliations:** ^1^ Department of Molecular Medicine, Faculty of Advanced Medical Sciences, Tabriz University of Medical Sciences, Tabriz, Iran; ^2^ Department of Clinical Biochemistry and Laboratory Medicine, Faculty of Medicine, Tabriz, Iran; ^3^ Tuberculosis and Lung Diseases Research Center, Tabriz University of Medical Sciences, Tabriz, Iran; ^4^ Rahat Breath and Sleep Research Center, Tabriz University of Medical Science, Tabriz, Iran; ^5^ Cellular and Molecular Biology Research Center, Shahid Beheshti University of Medical Sciences, Tehran, Iran; ^6^ Infectious and Tropical Diseases Research Center, Tabriz University of Medical Sciences, Tabriz, Iran; ^7^ Department of Medical Biochemistry, Faculty of Medicine, Istanbul Aydin University, Istanbul, Türkiye

**Keywords:** cell cycle, luminal breast cancer, chromosomal instability, copy number variation, therapeutic strategy

## Abstract

Breast cancer is the most prevalent neoplasm in women. ER^+^ (Luminal subtype), representing over 70% of breast tumors, is a genetically diverse group. Structural and Numerical-Chromosomal instability initiates tumor development and is recognized as the primary driver of genetic alteration in luminal breast tumors. Genomic instability refers to the increased tendency of cancer cells to accumulate genomic alterations during cell proliferation. The cell cycle check-point response to constant and stable genomic alterations in tumor cells drives this process. The impact of CNV patterns and aneuploidies in cell cycle and proliferation perturbation has recently been highlighted by scientists in Luminal breast tumors. The impact of chromosomal instability on cancer therapy and prognosis is not a new concept. Still, the degree of emerging genomic instability leads to prognosis alteration following cell cycle deregulation by chromosomal instability could be predicted by CNVs-based reclassification of breast tumors. In this review, we try to explain the effect of CIN in the cell cycle that ended with genomic instability and altered prognosis and the impact of CIN in decision-making for a therapy strategy for patients with luminal breast cancer.

## 1 Introduction

Carcinomas, malignant neoplasms of epithelial origin, are characterized by uncontrolled cell proliferation driven by the accumulation of genetic alterations (1, 2). Breast cancer, the most frequently diagnosed cancer worldwide (3, 4), is classified into four distinct subtypes based on immunohistochemical (IHC) and genetic profiling of estrogen receptor (ER), progesterone receptor (PR), and human epidermal growth factor receptor 2 (HER2) status. These subtypes include: Luminal A (ER^+^ and/or PR^+^, HER2^-^), Luminal B (ER^+^ and/or PR^+^, HER2^+^), HER2 positive (ER^−^, PR^−^, and HER2^+^), and triple-negative breast cancer (ER^−^, PR^−^, and HER2^-^). These subtypes exhibit distinct prognoses and responses to therapy (5–8), guiding clinical management to appropriate chemotherapy selection. To further refine IHC and fluorescence *in situ* hybridization (FISH), and overcome their limitation issues and cut-off challenges, the molecular signature of breast cancer subtypes is assessed by mRNA-based kit. These kits incorporate four novel proliferation markers (MKI67, PCNA, CCNA2, and KIF23), in addition to the three established biomarkers, providing a more comprehensive molecular profile for accurate subtyping and prognostication (9, 10). Notably, luminal subtypes constitute approximately 70% of breast cancers diagnosed in women, with 85% exhibiting 5-year overall survival. However, sub-classification for therapeutic purposes remains challenging due to the molecular diversity of ER^+^ and/or PR^+^ tumors (Table 1) (11–13).

**TABLE 1:** Comparison of classification methods of breast cancer.

Comparison of classification methods of breast cancer		
	Methods	Theranostic impact
Intrinsic subtyping (4 subtypes)	The early method was based on immunohistochemistry (ER, PR and HER2) (6)	• Low theranostic impact (119) • Poor prediction of late relapses (120)
	The new method is based on gene expression data	
Integrative clustering subtyping (10 subtypes)	This is based on copy number alteration (102)	• High theranostic impact (119) • Dynamics of late relapse provided (120) • Improved decision-making of adjuvant and neo-adjuvant therapy for ER^+^ subtypes (121, 122)

**FIGURE 1:**
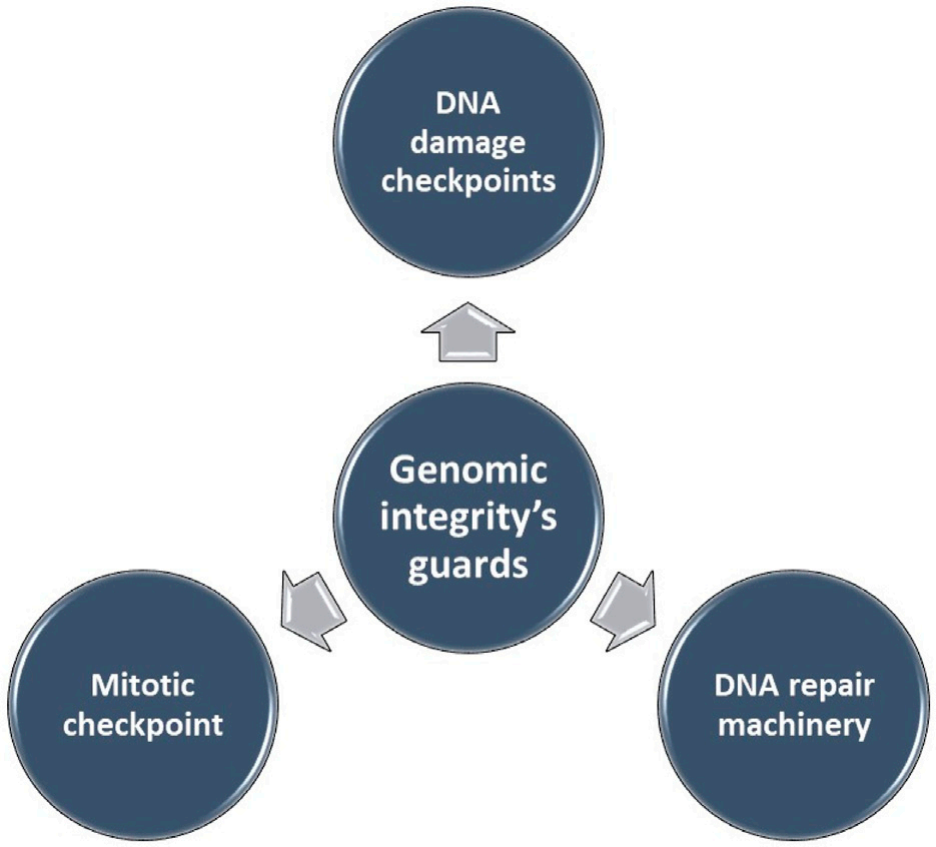
The maintenance of genomic integrity is safeguarded by intricate mechanisms.

Genomic instability (GI), the increased susceptibility of the genome to accumulate further alterations driving tumor clonal evolution and contributing to the heterogeneity observed in solid tumors. Genome copy number variations (CNVs), encompassing deletions, insertions, and duplications of variable-sized DNA segments, are the primary drivers of genomic instability in various cancers (14–16). The maintenance of genomic integrity, crucial for cellular function, is safeguarded by intricate mechanisms (Figure 1). Disruptions in these mechanisms can lead to genomic instability.

In breast cancer, several pathways crucial for maintaining genomic integrity are frequently altered, including some that have been successfully targeted for therapeutic intervention (15). The study of CNVs has undergone significant advancements since their initial discovery. Early research relied on techniques like array comparative genomic hybridization and SNP genotyping platforms, which enabled the identification of numerous CNVs across the human genome. With the advent of next-generation sequencing (NGS), the CNV research field has witnessed a dramatic shift. NGS offers a more comprehensive and high-throughput approach to identifying CNVs, enabling researchers to delve deeper into their genomic architecture and functional consequences (17). CNVs, a form of structural alteration characterized by variable-sized deletions, insertions, and duplications of DNA segments, represent some of the most prevalent and significant genetic alterations. Structural chromosomal instability (s-CIN), the underlying cause of CNVs in cancer, links cell cycle checkpoints, genomic instability, and mitogen-induced replication stress. Notably, CNVs in combination pathway analysis revealed that identical DNA gains encompass essential genes acting as tumorigenic drivers. The challenge lies in translating these findings into clinically relevant classifications. In breast tumors, integrative clustering based on CNVs, transcriptomic data, and clinical characteristics demonstrates the strongest correlation with survival and genomic instability development (18, 19). This approach, known as Integrative Clustering (IntClust 1-10), classifies breast cancer samples based on their genomic driver events. While luminal A and B subtypes may appear indistinguishable based on shared genomic characteristics (20), luminal B tumors exhibit greater heterogeneity in CNV patterns. This highlights the challenge of subtype classification, as tumors display a continuous spectrum of molecular formations associated with increasing levels of genetic damage (21).

This review summarizes the impact of CNVs on cell cycle behavior in the luminal subtype of breast cancer. We explore how CNVs independently or in conjunction with other factors alter the status of cell cycle checkpoints. Finally, we provide novel insights into the implications of CNVs for clinical therapeutic decision-making.

## 2 Literature review methodology

This review employed a comprehensive search strategy to identify relevant literature from reputable scientific databases, including Science Direct, Springer, RSC, ACS, NCBI, MDPI, Web of Science, and Google Scholar. The inclusion criteria were as follows: full-text articles published between 1994 and 2024, articles published in the English language, articles with a title, abstract, source, DOI, and year of publication.

Keywords such as “Genomic Instability in Cancer”, “Cell Cycle Deregulation in Cancer”, “Breast Cancer Subtypes”, and “Luminal Breast Cancer Genetic Variations” were used and combined with search terms using Boolean operators to refine the search. Full-text unavailable articles, non-English language, or summaries of editorials, conferences, seminars, and events were excluded.

## 3 Copy number variation sources and analysis

Principally, CNVs as a somatic alteration derive from several genetic mechanisms, especially gene instability, chromosomal instability (CIN), and aneuploidy, which contribute significantly toward the development and prevalence of CNVs observed in luminal breast cancers (22). The most impactful aspects of CNVs in luminal breast cancer are oncogene amplification (epithelial membrane protein 3) (23), prognostic value (phorbol-12-myristate-13-acetate-induced protein 1) (24), molecular subtyping (human epidermal growth factor receptor 2) (25), personalized treatment (Myeloid differentiation primary response 88) (26), biomarker (PGAP3, GRB7 genes) (27), and drug resistance mechanisms (MYC gene regulation) (28).

Genomic instability results from defects in several mechanisms during cell cycle progression and regulation. These mechanisms include DNA damage from both endogenous and exogenous sources, DNA damage repair, DNA replication and transcription, mitotic chromosome segregation, centrosome amplification, epigenetic modifications, and telomere maintenance. The GI can either be perpetuated or limited through the induction of mutations or aneuploidy, both of which can be enabling or catastrophic in their effects. GI is closely linked to immune evasion, cancer progression, and multidrug resistance. In hereditary cancers associated with microsatellite or chromosomal instabilities, mutations in DNA repair genes are primary drivers of GI. However, in sporadic cancers, particularly in their early stages, GI does not typically depend on mutations in DNA repair or mitotic checkpoint genes. The mutation patterns observed in sporadic cancers suggest that p53 mutations are driven by DNA damage, rather than through activation of p14ARF. Oncogene-induced DNA damage may represent a critical contributor to GI in sporadic cancers (14, 29–32).

**FIGURE 2:**
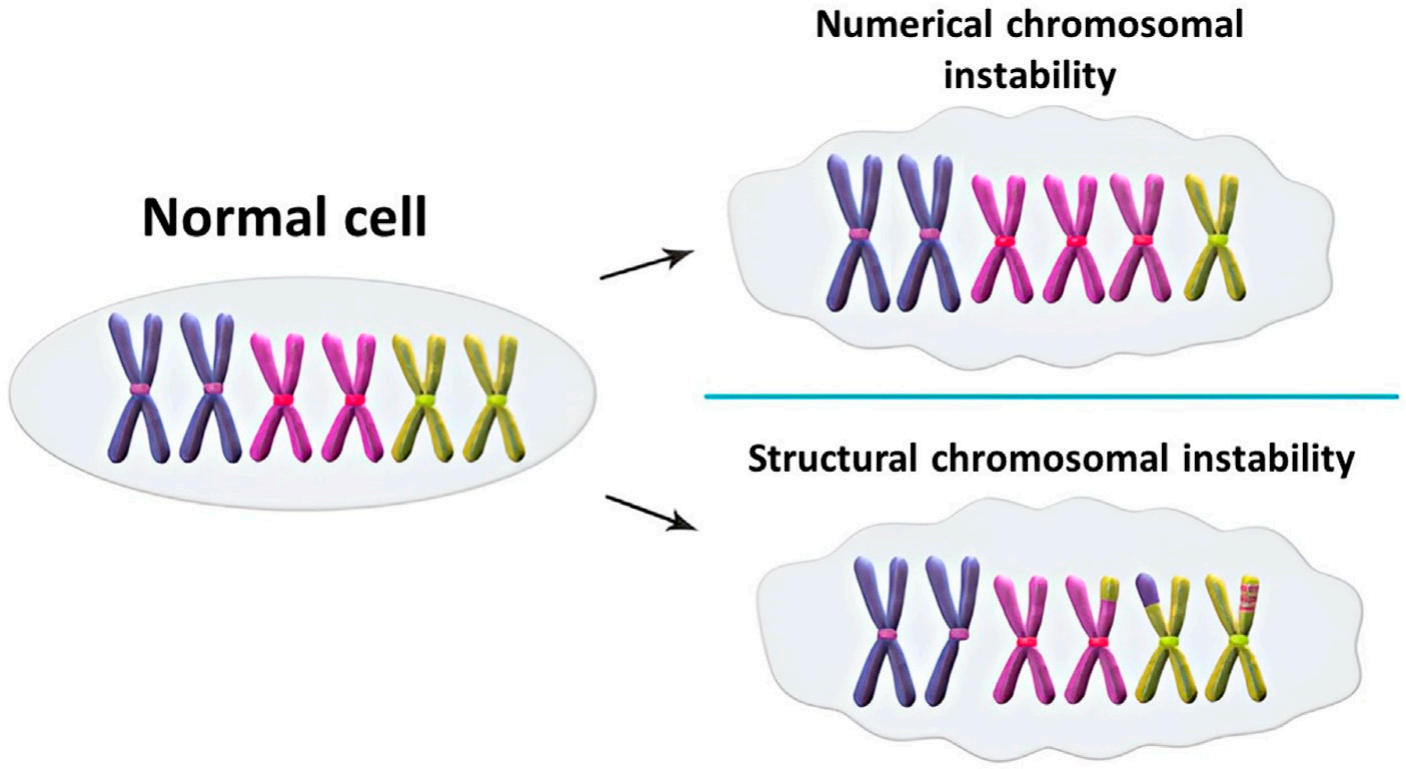
Numerical and structural chromosomal instability (CIN) (15).

Maintaining genomic stability necessitates the precise replication of genetic material and the equal segregation of newly duplicated chromosomes during mitosis. Disruptions in either of these processes can lead to chromosomal instability (CIN), characterized by an increased rate of chromosomal alterations (33). These chromosomal alterations serve as unique fingerprints, unveiling the intricate mechanisms by which genes become dysregulated in cancer, leading to the destabilization of the cell’s genetic integrity (34). CIN manifests as either numerical (n-CIN) or structural (s-CIN) alterations (Figure 2). n-CIN involves gains or losses of whole chromosomes, while s-CIN includes gains, losses, or rearrangements of chromosome segments. n-CIN primarily results from mitotic errors, whereas s-CIN arises from pre-mitotic events affecting chromosome integrity (33).

CNVs refer to variations in the copy number of specific DNA segments across different individuals’ genomes (35), approximately 200 CNVs have been identified breast cancer (36). CNVs contribute significantly to genomic diversity, varying from small alterations to complete chromosomal aneuploidies. While small CNVs are usually benign, larger ones (over 250 kb) are linked to serious consequences like developmental disorders and cancers (37). Analysis of the top 100-upregulated genes associated with various cancers reveals distinct chromosomal locations. Table 2 summarizes the findings of studies on CNVs in various types of cancer. Such validation could pave the way for the development of more effective diagnostic, prognostic, and therapeutic strategies, ultimately leading to a more promising future in the fight against cancer (34). Various techniques have been employed for CNV profiling, including SNP arrays and next-generation sequencing (NGS) technologies. Ongoing efforts are focused on developing more accurate and efficient methods for CNV detection, as well as exploring the functional implications of CNVs in biological processes and disease contexts (17). CNV holds significant promise as a valuable biomarker for tumors, with potential applications in tumor subtyping, drug response prediction, and survival time prediction (38). Targeted NGS-based CNV panels are widely used in clinical practice due to their high reproducibility and focus on a limited gene set, making them crucial for accurate clinical decision-making, particularly in cancer treatment. Insights from genome-wide CNV studies aid in designing these targeted panels. However, normal cell contamination, tumor heterogeneity, and aneuploidy hinder reliable CNV detection. Additionally, systematic errors from structural variations can bias results, making it advisable to detect these variations before conducting CNV analysis on tumor samples (38). Single-cell sequencing presents challenges for CNV analysis due to limited DNA content, making it difficult to differentiate between PCR duplication. Accurate CNV measurements become unreliable with restricted DNA quantities in single cells and circulating tumor DNA, necessitating careful adjustments for proper analysis. Additionally, integrating CNV data with other multi-omics information is essential for a comprehensive understanding of cancer development and progression (38).

**TABLE 2:** Significant CNVs in various cancers.

Cancer type	Chromosomal structural changes	Genomic instability rates	Ref.
Endometrial Cancer	Mainly located in 8q and 1q. Includes CCNE1, ERBB2, KRAS, MYC, PIK3CA genes Few aneuploidy changes in Type I cancers; a greater number in Type II (high grade) and advanced FIGO stage	Low in Type I High in Type II and advanced stages	(123)
Non-small cell lung cancer	Includes ACOT1, NAA60, GSDMD, SLC35B3, HLA-DPA1 genes	Moderate	(124)
Hepatocellular carcinoma	Chromosomal changes across 1-22. Contains 13,839 CNVs with 138 CNV-driven genes (93 duplicated, 45 deleted) Highest duplication and deletion were observed on chromosomes 1, 5, and 4 NPM1 gene implicated	High	(125)
Colorectal cancer	Amplifications observed on 1q, 8q, and 5q involving cell cycle, DNA repair, and WNT signaling pathways genes Deletions found on 1p, 4q, 8p, 17p, 18q, and 22q	Low in stage II Moderate in stage III High in stage IV	(126)
Breast and Prostate Cancer	Common SCNAs (Somatic Copy Number Alterations) identified, and associated with disease recurrence and survival	Variable	(127)
Breast Cancer	CNV-related genes identified with prognostic significance	Moderate	(128)
Ovarian Cancer	CNVs at known risk alleles associated with increased ovarian cancer risk	High	(129)
Multiple Cancers	Various chromosomal structural changes Includes 523 identified CNVs	Variable	(130)

HCC, hepatocellular; Ch, chromosome; WNT, Wingless-related integration site; CRC, colorectal cancer; P, polyps; H.C., healthy controls.

Aneuploidy is an abnormal number of chromosomes in a cell, deviating from the typical diploid number. For instance, a human cell with 45 or 47 chromosomes instead of the usual 46 would be considered aneuploidy (39). Aneuploidy manifests in two distinct forms: stable and unstable. Stable aneuploidy exhibits consistent numerical chromosome alterations across most cells, while unstable aneuploidy is characterized by significant cell-to-cell variation in chromosome number, leading to karyotypic heterogeneity. Unstable aneuploidy notably promotes the development of diverse tumor subpopulations, contributing to both inter and intra-tumoral genomic heterogeneity. CIN is a major contributor to intra-tumoral heterogeneity, which empowers cancer cells to adapt to environmental pressures and evolve into more aggressive, treatment-resistant populations. CIN and aneuploidy profoundly affect therapeutic response by disrupting gene regulation and altering protein levels, ultimately influencing cellular responses to drug treatments. This dynamic interplay suggests that individual cancer cells with distinct chromosomal profiles may exhibit varying drug sensitivities, potentially allowing a subset of the tumor population to evade therapy and persist. A pilot study on ten Luminal B patients suggests an association between stable aneuploidy, intermediate CIN, and lympho-vascular invasion. This association between clonal heterogeneity and CIN holds potential prognostic value for breast cancer patients, particularly those with HER2^+^ tumors. Dual-color FISH analysis of Luminal B subtype breast cancer revealed that over 90% of patients exhibited intermediate CIN (33%-49%), while only 10% showed high CIN (52%) (40). Aneuploidy Score (AS), a measure of CIN, indicated that 75% of breast cancers had an AS above five. Luminal A tumors had lower AS compared to Luminal B. While AS was not prognostic for overall survival, progression-free survival was significantly worse in Luminal B cancers with high AS. The most frequent chromosome gains were observed in 1q, 8q, and 16p, while losses were commonly seen in 16q, 17p, and 8p (41). Notably, centromere 17 copy number, assessed by FISH and NGS-derived for CIN scores, emerged as a key prognostic factor in breast cancer (40, 42). Furthermore, Phosphatase and TENsin homolog (PTEN), a tumor suppressor gene frequently altered in breast cancer, exhibits distinct clinical characteristics compared to other cancers. Women with PTEN hamartoma tumor syndrome have an up to 85% lifetime risk of breast cancer. This alteration was identified by integrating somatic CNV and transcriptome data (43). Consequently, traditionally approved methods for CNV calling from tumor samples have demonstrated limited reliability for these proposes. Validation of these traditional methods in advance, MLPA or array-based approaches have better resolution (44).

A recent article about the reliability of DNA copy number variation linked machine learning approach CopyClust for integrative cluster classification (IntClust) for breast cancer. Particularly breast cancer subtyping is highly correlated to genomic variations of tumor, and CNVs are highly mirrored with cancer-driving genes additionally specific CNVs reflect tumor behavior and development. So IntClust classification could achieve 81% success on TCGA SNP data and 79% on WES data. Another, valuable advantage of the CopyClust algorithm is the ability to process CNV data alone when transcriptomic data is useless or not accessible. CopyClust classification has maximum clinical outcome prediction power with high accuracy (45). Table 3 comprises the traditional and updated methods in structural chromosomal variation detection in tumor samples.

**TABLE 3:** Comparison of Traditional Methods versus updated methods in structural chromosomal variation detection in tumor samples.

Method	Resolution	Genome-wide coverage	Key applications	Limitations	References
Karyotyping	Low (>5–10 Mb)	No	Large chromosomal abnormalities	Limited resolution	(131)
FISH	High (targeted)	No	HER2 amplification, specific loci	Not genome-wide	DOI: 10.1126/science.1586340
Standard CGH	Low (>5 Mb)	Yes	Large CNVs	Labor-intensive, low-resolution	(132)
aCGH	High (10–50 kb)	Yes	CNVs across the genome	Expensive cast, low-resolution power in rearrangement detection	DOI: 10.1073/pnas.89.24.11538
qPCR	High (targeted)	No	Targeted amplifications	Not genome-wide, limited targets	DOI: 10.1007/s00335-006-0045-9
MLPA	High (targeted)	No	Targeted CNV detection	Limited to predefined loci	(133)
SNP Arrays	Moderate	Yes	SNP and CNV analysis	May miss poorly covered regions	DOI: 10.1101/gr.6021207
NGS CopyClust	High	Yes	Any variations in the genome	Expensive cast	DOI: 10.1126/science.1100226

FISH: fluorescence *in situ* hybridization, Standard CGH: array Comparative Genomic Hybridization, aCGH: array comparative genomic hybridization, qPCR: Quantitative PCR, MLPA: multiplex ligation dependent probe amplification, SNP, Arrays: Single Nucleotide Polymorphism Arrays, NGS: Next-Generation Sequencing).

### 3.1 Comparison of traditional methods versus updated methods in structural chromosomal variation detection in tumor samples

Traditionally approved methods for CNV calling from tumor samples did not perform well to ensure the best reliability for this purpose. Validation of these traditional methods in advance, MLPA or array-based approaches have better resolution. For multiple samples prevalent CNVs called is highly recommended (44). A recent article about the reliability of DNA copy-number-variation linked machine learning approach CopyClust for integrative cluster classification (IntClust) for breast cancer.

Particularly breast cancer subtyping is highly correlated to genomic variations of tumors, CNVs are highly mirrored with cancer-driving genes additionally specific CNVs reflect tumor behavior and development. So IntClust classification could achieve 81% success on TCGA SNP data and 79% on WES data. Another, valuable advantage of the CopyClust algorithm is the ability to process CNV data alone when transcriptomic data is useless or not accessible. CopyClust classification has maximum clinical outcome prediction power with high accuracy (45).

### 3.2 Genomic changes and aneuploidy in normal breast tissue

Recent studies indicate that Aneuploidy could be observed in non-cancerous normal breast tissue. The link between normal breast cells aneuploidy, certain breast cancer types, and age is notable.

A key study in the context of single-cell DNA sequencing from normal breast tissue samples of 49 healthy women. With a middle rate of 3.19% aneuploidy could be determined in the study group. These cells had changes like gain of chromosome 1q and incomplete loss of 10q, 16q, and 22q, which are characteristic chromosomal structural variations in invasive breast cancers. In particular, the prevalence of aneuploid cells elevated with age shows the possibility of natural accumulation of chromosomal instability over time (45). Non-cancerous events linked to aneuploidy in luminal cancers include: Differentiation Maintenance: Aneuploid cells control differentiation-correlated genes, like those in estrogen receptor pathways, preserving the luminal type breast cancer (DOI: 10.1038/bc2009). Cell Cycle Checkpoints: p53 activation in response to handling replication stress, may lead to more genomic stability (46). Dormancy or Senescence: The entrances of aneuploid cells to non-proliferative states, improve tumors genomic stability without invasion (47).

The possible Mechanisms of Aneuploidy Maintenance In normal tissues, without fostering tumorigenesis include Chromosomal Instability Tolerance: Autophagy and proteasomal degradation manage stress caused by aneuploidy (48), SAC^1^ Regulation: Fine-tuned SAC signaling stabilizes chromosomal integrity (DOI: 10.1038/nrm.2011.10) and Epigenetic Regulation: Histone modifications and DNA methylation help stabilize gene expression profiles (49).

In summary, it is widely accepted that Aneuploidy in normal breast tissue is an age-age-correlated event that is a normal consequence of early genomic instability unset. While some aneuploid cells harbor the same chromosomal alterations of luminal subtypes of breast cancers, their insignificant proliferation rate increases and non-tumorigenic mechanisms suggest self-limiting phenomena, arise from positive selection in tumor evolution. However, the influence of these cells on cancer development is not fully understood.

## 4 Cell cycle

The cell cycle, a tightly regulated sequence of events culminating in cell division, ensures the faithful transmission of genetic material from parent to daughter cells. This intricate machinery relies on the coordinated action of cyclins and CDKs^2^ throughout distinct phases: G0, G1, S, G2, and mitosis. Embedded within the cell cycle are five distinct checkpoint switches, which, according to mathematical models, may operate in a bistable manner. Activation of the G1/S restriction checkpoint and the spindle assembly checkpoint (SAC) in the G2/M transition elicits unique responses, whereas activation of the S and G2 checkpoints triggers diverse molecular outcomes. Notably, experimental validation has only confirmed the bistable switch property of the restriction checkpoint (50, 51).

### 4.1 G0 and G1 phases

In contrast to the quiescent G0 phase, extensive mRNA and protein synthesis, leading to cell growth and preparation for DNA replication, characterize the G1 phase. Mitogenic signaling pathways play a crucial role in G1 progression, particularly in epithelial cells (52–54). Intracellular and extracellular mitogenic signals activate the Cyclin D/CDK4/6 complex, resulting in the mono-phosphorylation of the RB^3^ within the E2F1/RB complex. Subsequently, the Cyclin E/CDK2 complex further phosphorylates RB, leading to the complete release of the E2F transcription factor. Active E2F1 drives the transcription of genes essential for DNA replication in the S phase. The G1/S checkpoint, also known as the restriction checkpoint, is the first critical control point in the cell cycle. This checkpoint regulates cell cycle progression by monitoring critical conditions, including growth factor availability, nutrient levels, and cell size. If these conditions are unfavorable, the cell cycle arrests in G1, returning to the G0 state. The balance between G1/S checkpoint activity and proliferation stimuli, along with the presence of the cyclin-dependent kinase inhibitor 1A (P21), determines the population of cells in G0 (55). Successful passage through this checkpoint signifies the cell’s irreversible commitment to division (51, 56).

Recent studies have highlighted the involvement of CDK2 in apoptosis regulation, DNA and RNA metabolism, and interaction with p53 in tumor development. Ectopic CDK2 expression has been linked to ductal breast carcinoma pathogenesis, with high nuclear CDK2 levels correlating with aggressive phenotypes, particularly high tumor grade, and lymph vascular invasion (57). Additionally, CDK4/6 plays a critical role in the luminal subtype of breast cancer. Analysis of 320 elderly metastatic breast cancer patients with luminal A and B subtypes revealed that CDK4/6 inhibitors facilitate treatment (58).

### 4.2 Deregulated G1 phase and restriction checkpoint in luminal breast cancer

CNV patterns exhibit a strong correlation with proliferation. The interplay between the G1/S checkpoint and E2F activity is modulated by mitogenic upstream signals, such as HER2 activation and Cyclin D1 expression levels. Studies on luminal breast tumors have demonstrated that CNVs impact the functional status of E2F as a transcription factor (59). CNVs regulate the phosphorylation-dependent release of E2F from the RB-E2F complex, leading to three distinct phenotypes in response to oncogene activity: G1 arrest, G1 acceleration, and G0 quiescence (60). Table 4 contains the specific genes with altered copy numbers that regulate the phosphorylation-dependent release of E2F from the RB-E2F complex.

**TABLE 4:** The specific genes with altered copy numbers regulate breast cancer luminal subtypes associated with the phosphorylation-dependent release of E2F from the RB-E2F complex.

Chromosomal region	Gene(s)	Alteration type	Frequency in luminal A	Frequency in luminal B	Associated function	Genomic instability	Impact on CDK4/6 inhibitor response	Ref.
1q21-q44	MCL1, BCL9	Amplification	25%–40%	50%–65%	Promotes cell survival and proliferation; linked to poor prognosis.	Enhance chromosomal instability and resistance to apoptosis	Potential resistance is due to promoting survival pathways	(134)
8q24	MYC	Amplification	20%–30%	40%–50%	Oncogene amplification; drives cell proliferation and metabolic reprogramming.	Elevated replication stress and mutational burden	May cause resistance through increased proliferation	(135)
11q13	CCND1 (Cyclin D1)	Amplification	40%–50%	60%–75%	Control cell cycle progression; highly amplified in Luminal B subtype	Enhance chromosomal instability and cell cycle deregulation	Amplification linked to resistance Over-expression may lead cell cycle developing independent of CDK4/6 inhibition	(136)
17q12	ERBB2 (HER2)	Amplification	<5%	15%–20%	HER2 amplification is more frequent in Luminal B than Luminal A, causing invasive tumor behavior	Enhanced genomic instability due to aberrant proliferation signaling	HER2 amplification may lead to resistance; mix therapies targeting both pathways are under study.	(137)
16q22.1	CDH1	Deletion	20%–30%	40%–50%	Loss of E-cadherin, damage cell adhesion and encourage metastasis	Enhance chromosomal repositioning and metastatic power	The effect on CDK4/6 inhibitor response is under investigation	(138)
13q14	RB1	Deletion or Mutation	10%–15%	30%–40%	Tumor suppressor loss; stimulates cell cycle deregulation and genomic instability	Deregulate cell cycle checkpoints, increased mutational burden	Loss-of-function (LOF) mutations link to resistance; RB1 is pivotal for CDK4/6 inhibitor efficacy	(139)
8p21-p23	LZTS1, DLC1	Deletion	10%–20%	25%–35%	Loss of tumor suppressor genes; link with enhanced metastatic potential.	Decrease genome perfection and enhanced metastasis	The connection on the CDK4/6 inhibitor answer is under study	(140)
20q13.2	ZNF217	Amplification	15%–25%	30%–40%	Lead to tumor progression and resistance to apoptosis	Elevate genomic instability, and stimulate tumor evolution	Potential resistance unset due to stimulating survival pathways	(141)
17p13.1	TP53	Deletion or Mutation	10%–15%	30%–50%	Loss of the TP53 tumor suppressor is frequently detected in aggressive Luminal B	Massive genomic instability due to damaged DNA repair	TP53 mutations may lead to resistance Update studies are ongoing	(142)
7q21.2	CDK6	Amplification	Variable	Variable	CDK6 amplification can promote resistance to CDK4/6 inhibitors by scape CDK4 inhibition	Increased cell cycle progression and potential resistance	Amplification related to resistance; CDK6 overexpression can decrease sensitivity to CDK4/6 inhibitors	(143)
12p13.33	CCNE1 (Cyclin E1)	Amplification	Variable	Variable	Cyclin E1 stimulates cell cycle passage from the G1 to S phase	intensify genomic instability due to uncontrolled cell cycle entry	Amplification associated to resistance; overexpression may force cell cycle progression-free of CDK4/6 inhibition.	(144)

Structural chromosomal aberrations, such as CNV patterns, emerge early in tumor evolution and drive tumor progression. CNVs affect large chromosomal segments, some of which harbor known oncogenes. Depending on the proliferation potential and expression dosage, CNVs can alter the G1 phase function. Additionally, tissue-dependent expression of G1 phase inhibitors plays a role (61). Interestingly, a cancerous cell with identical oncogene expression can exhibit two distinct G1 phenotypes, depending on the E2F bistable switch status (55). Biological bistable switches are ultrasensitive biochemical systems that exhibit two distinct activation states: low and high. These switches arise in systems with multiple inhibitors and activators that create negative and positive feedback loops. A well-known example is E2F activation, which can exist in high or low levels despite steadily increasing mitogen signals. This allows the switch to be either ON or OFF (62–65).

**FIGURE 3:**
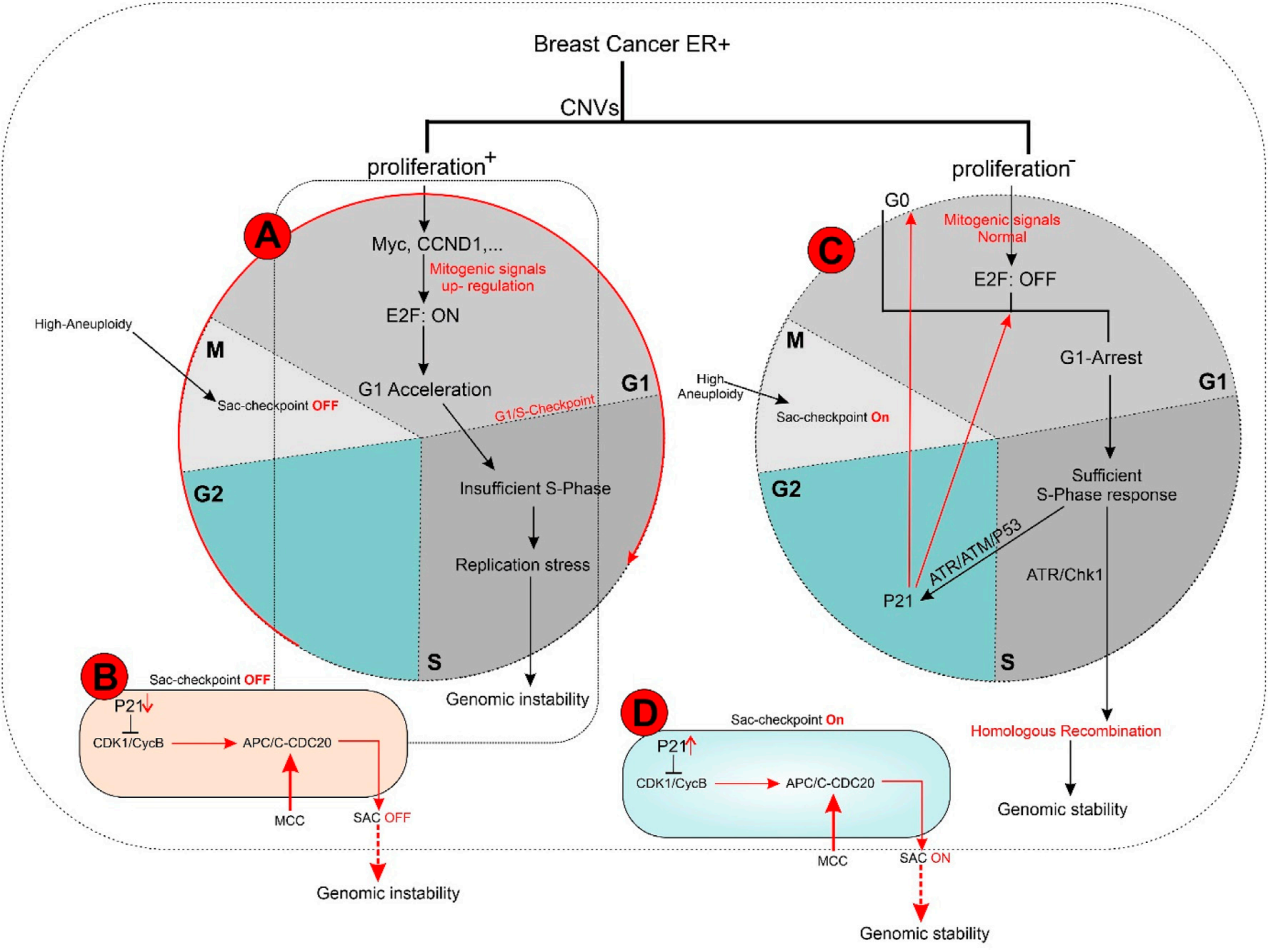
**(A, B)** In ER + breast tumors, oncogene overexpression due to identical CNVs leads to an increased proliferation rate by aberrant E2F release and accelerated G1 phase. An E2F ON status eradicates complete protein transcription in the G1 phase, which is necessary for replication stress defense in the S phase and limits response capacity to DNA damage. In turn, improper S phase checkpoint activation leads to a limited release of P21 as soon as G2 transcription activity initiation. A decreased concentration of P21 restricts its inhibitory effect on CDKs. In next G1, the lack of P21 leads to aberrant activation of CDK4 and CDK2 that in complex with specific cyclin proteins cause massive E2F activation. In the immediate M phase induction, CDK1 in CDK1/CycB complex leads to activation of APC/C-CDC20 independent of SAC inhibition. This series of molecular events initiated with identical CNVs results in a high proliferation rate and progression genomic instability due to the limited capability of DNA repair mechanisms. Additionally, SAC checkpoint’s improper function leads to sustain aneuploidy during tumor progression and is terminated with genomic instability. **(C, D)** In conditions where oncogene content does not reach E2F bistable capacity, the S phase displays the optimal response to DNA damage leading to P21 increased levels of transcription during G2 by activation of ATR/ATM/P53 pathway. P21 inhibitory effect on CDKs plays a critical role in the development of G0 or G1 arrest in the next cell cycle by inhibiting CDK4 and CDK2. On the other hand, upregulation of P21 in the M phase inhibits CDK1/CycB by blocking CDK1 leading to the dependency of APC/C-CDC20 function on MCC in the SAC checkpoint. Result resistance to progression aneuploidy in tumor cells. This chain of molecular events results in a low proliferation rate, low genomic instability, and insignificant aneuploidy progression.

In the E2F: OFF position, the G1 phase arrests cells based on P21 and E2F functional levels in an identical clonal population, containing variable G0 cells. Tumor masses often contain a significant population of G1-arrested and G0-phase cells, which dramatically limits the proliferation rate. This allows cells in the S phase to focus protein synthesis on DNA damage repair and replication stress defense (Figure 3) (55, 66).

In the E2F: ON position, G1-accelerated tumor cells exhibit a short G1 phase due to increased expression of direct and indirect mitogens (e.g., Myc and HER2) or direct E2F activators (e.g., cyclin D1 and cyclin E2) as G1 kinase complexes. Accelerated G1 leads to elevated proliferation rates, resulting in two undesirable consequences:

1. Linear tumor evolution: A single or limited number of clones can become dominant within a tumor mass due to their rapid proliferation, gaining an evolutionary advantage. This selected clone provides a suitable environment for accumulating mutations that enhance the fitness of cells to the tumor microenvironment.
2. Genomic instability: The shortened G1 phase compromises the S-phase response mechanisms, leading to genomic instability and hyper-mutation, which fuel tumor progression. This instability occurs due to elevated E2F levels, which increase cyclin E2 production while reducing the synthesis of ATR/ATM response machinery. ATM/ATR is crucial for ongoing DNA damage repair and replication stress defense, processes limited by the short G1 phase (18, 66, 67). E2F1 regulates some genes in the ATR/ATM pathway but does not fulfill all the requirements for the S-phase checkpoint machinery. Consequently, an accelerated G1 phase cannot adequately support the S-phase response to replication stress. However, as long as the G1 phase remains active, the luminal tumor can still generate sufficient factors for an appropriate S-phase response.

### 4.3 S phase

During the S phase, the cell duplicates its genetic material and prepares for division into two daughter cells. Activation of the CycA/CDK2 kinase in the early S phase triggers the removal of CDC6 from the ORC^4^, initiating DNA replication (50, 51). The S phase checkpoint is a complex system involving ATM^5^, ATR^6^, and DNA-PKcs^7^ kinases. These kinases act as evolutionary sensors for DNA damage, which can lead to structural malformations like DSBs^8^ or SSBs^9^ during replication.

Following DNA damage, ATR/ATM activates distinct pathways. In response to replication stress-induced single-stranded tails, the resection of DSB ends activates ATR/CHK1^10^. Notably, ATR, not ATM, plays a crucial role in responding to replication stress arising from DNA breakage or TRCs^11^, which generate RNA: DNA hybrids (33, 56). While the G1/S checkpoint converges to a single response based on E2F activation status in response to diverse oncogenic stimuli, the S-phase checkpoint activation relies on a limited number of sensor molecules but generates divergent responses with varying intensities. These S checkpoint-dependent compensatory responses, which tend to spread across different cell cycle phases, ultimately lead to perturbations throughout the entire cell cycle (Figure 3) (68).

### 4.4 G2 phase

During the G2 phase, cellular DNA content reaches 4N, and protein synthesis resumes. Newly synthesized proteins, including those regulated by the FOXM1^12^ transcription factor, play a crucial role in the subsequent phases of the cell cycle. G2 phase progression is driven by the CycA/CDK1 and CycB/CDK1 kinase complexes, which activate BORA^13^. Bora, in turn, activates Aurora-A, a key regulator of M phase entry, marking the “commitment to mitosis” (66). The G2/M transition checkpoint relies primarily on the CycA-CDK1 and CycB-CDK1 complexes, working in tandem. This checkpoint is sensitive to DNA damage, utilizing ATR/ATM sensory molecules and their partners CHK1/2^14^. However, the “checkpoint nature” of the G2/M checkpoint is debatable due to its relatively fixed duration, even in response to ATR/ATM activation (68, 69).

### 4.5 Deregulation of the S and G2 phase and related checkpoints in luminal breast cancer

The S and G2 phases are crucial for ensuring the error-free replication of a cell’s genetic material. These checkpoints rely on the sensory machinery of ATR/ATM and DNA-PK molecules. While each sensor has a distinct function and activates different molecular targets, ATR/ATM activation plays a particularly influential role in the entire interphase by triggering multiple pathways. DNA damage, including DSBs and SSBs generated during DNA replication, is detected by ATR and S checkpoint sensors. A multitude of endogenous and exogenous factors can induce DNA damage in cancer. In luminal-type breast cancer, the primary source of genomic instability stems from the S phase checkpoint’s response to persistent replication stress, which can arise from various conditions, including stalled replication forks, fragile site prevalence, and oncogene overexpression (56, 70). Replication stress can arise from various conditions, including the prevalence of fragile sites -chromosomal breakpoints- and oncogene overexpression. These factors can lead to stalled replication forks. Notably, the breakpoints of CFSs^15^ are often located in regions of RNA: DNA hybrid zones. This change in DNA topology induces replication-transcription conflicts, which are the primary cause of single-stranded DNA fragment formation (71). The S phase checkpoint primarily relies on ATR/CHK1 rather than ATM/CHK2 to resolve replication stress (72, 73). In the context of altered DNA integrity caused by high E2F levels, the cell’s DNA damage repair capacity may exceed the threshold of ATR/CHK1, which plays a central role in this process. Consequently, the cells become unable to effectively repair DNA damage using mechanisms like HR^16^ (33, 56).

Studies have shown a correlation between the frequency of chromosomal breakpoints and poor prognosis in luminal B breast cancer. Additionally, a subset of luminal A tumors with oncogene overexpression that rapidly exit G1 exhibit high genomic instability. This suggests that an arrested G1 phase may facilitate proper S checkpoint activation, highlighting the importance of sufficient time for repair to ensure genomic stability (71). The convergent nature of the ATR/CHK1, ATM/CHK2, and p53^17^ cascades allow for a coordinated response to replication stress in the S phase, leading to G1 and G2 arrest through the co-activation of distinct compensatory and repair pathways. For instance, p53 induces P21 expression in early G2, impacting the subsequent G1 phase by extensively inhibiting CDKs (Figure 3) (55, 74).

### 4.6 Mitosis

The mitotic phase, the microscopically visible portion of the cell cycle, is divided into five distinct stages. While there are no clear molecular markers for the transition from G2 to M phase, the reduction of Bora protein levels may indicate the initiation of spindle assembly. The progression of M phase is tightly regulated by two checkpoints: the SAC and the mitotic exit checkpoint (68). The SAC ensures the proper attachment of spindle fibers to chromosome centromeres during anaphase. Its function relies heavily on the regulation of the APC/C^18^ by CycB/CDK1. Active APC/C-CDC20^19^ triggers the conversion of separase (75).

#### 4.6.1 M phase dysregulation

The SAC ensures the proper attachment of spindle fibers to chromosome centromeres before anaphase, preventing the segregation of replicated chromosomes until each one is correctly connected. Deregulation of the SAC leads to aneuploidy, a frequent genomic alteration in solid tumors, including luminal breast cancer (33). Aneuploidy, also known as n-CIN^20^, correlates with genomic instability, chromosomal breakpoint frequency, and patient prognosis in luminal-type breast cancer (76). Aneuploid modifications are particularly pronounced in low-grade, early-stage tumors and remain remarkably stable throughout tumor development.

However, CINs and aneuploidy are distinct events, and the frequency of diploid/CIN^−^ and aneuploid/CIN^+^ status differs between luminal A and luminal B subtypes. Notably, 81% of Luminal B (HER2^+^) tumors are aneuploid/CIN^+^, while only 27% of Luminal A tumors exhibit this phenotype. This variation may be attributed to differences in proliferation capacity, which is highly correlated with the onset of aneuploidy (77). Aneuploidy (n-CIS) is frequently observed in luminal B and a subset of luminal A tumors with high oncogene expression (66, 78).

The SAC checkpoint relies on distinct sensors and activators to ensure accurate chromosome segregation. Its M phase restriction is highly dependent on the phosphorylation of APC/C-CDC20 by the MCC complex under normal growth conditions. The SAC checkpoint’s bistable character arises from a double-negative feedback loop involving CycB/CDK1 and the MCC complex. In the OFF state, following complete chromosome alignment on kinetochores, the MCC complex is degraded, leading to the formation of APC/C-Cdc20, which is activated by CycB/CDK1. This activation triggers the degradation of securin and Cyclin B, enabling successful chromosome separation. Conversely, when the SAC is in the ON state, unattached kinetochores provide a signal to block APC/C using the MCC complex (56, 75, 79–81). Recent studies have provided a deeper understanding of the SAC machinery and highlighted the regulatory role of CycB/CDK1. While CDK1 levels remain relatively stable throughout the cell cycle, Cyclin B expression increases during the metaphase-anaphase transition due to the activity of FOXM1-MuvB transcription factors. CycB/CDK1 initiates the activation of APC/C-CDC20, followed by its rapid degradation by active APC/C-CDC20. Interestingly, CycB/CDK1 also regulates its transcription factor, FOXM1-MuvB (68, 82). Previous studies have demonstrated the significant inhibitory effect of P21 on CDK4, CDK2, and CDK1 in the G1, G2, and M phases (83). P21 is regulated by P53, the effector molecule downstream of the ATR/ATM response to DNA damage-induced stress, including (CIN). Therefore, the ATR/ATM/P53 pathway can influence cell cycle transitions through P21’s function. Recent studies indicate that P21’s inhibitory effect on multiple cell cycle kinases, particularly those crucial for G1 and M phase checkpoints, exhibits a bistable switch property (66, 83). The balance between the SAC: OFF and SAC: ON states depends on oncogene activity and/or the frequency of n-CINs in tumors, influencing SAC checkpoint dynamics and tolerance versus stable aneuploidy. This balance could be targeted by anti-SAC agents (75, 84). These findings suggest that ER^+^ breast tumors exhibit a range of molecular diversity. The presence of identical CNVs and consistent cell cycle performance suggests potential therapeutic opportunities. This is based on the default aneuploidy that arises early in luminal tumor development and remains remarkably stable throughout tumor evolution.

## 5 Genetic alterations responsible for cell cycle checkpoint dysregulation in luminal-type breast cancers

Cancer classification based on driver genetic alterations reveals two major classes: “mutation driver” and “copy number variant driver.” Breast cancer is a prime example of the CNV-enriched class (85). In breast cancer, the primary driver of genomic alterations is consistent structural chromosomal and numerical aberrations. These CNVs frequently coincide with genes identified as neoplasm drivers, highlighting their functional significance. Notably, over 85% of genetic alterations shared by luminal subtypes are stable CNVs, occurring early in tumor progression and remaining remarkably stable throughout tumor evolution (86, 87). Their stability implies they are selected during the tumor’s evolution due to their role in providing growth advantages.

In the luminal A subtype, certain genetic changes tend to occur consistently. These include: Stable amplification (increased copies of genes): 1q region: for example, PIK3C2B and MCL1 genes, 8q region: such as the MYC gene, 16p region: including the FUS and DOK2 genes. Stable deletions (loss of genetic material): 16q region: for example, the loss of CDH1, which is associated with the loss of E-cadherin (a protein important for cell adhesion), 11q region: including the loss of ATM or CENPF genes.

Some specific stable CNVs that transpire in the luminal B subtype are amplification and deletion alteration. The main amplification of stable copy number variations is 17q (e.g., ERBB2, GRB7), 11q13 (e.g., CCND1, involved in cell cycle regulation), and 8q24 (e.g., MYC). Moreover, important deletions of stable copy number variations in luminal B are 13q (e.g., RB1 loss, contributing to increased proliferation) and 11q23 (ATM loss, impacting DNA repair) (27, 88).

In ER^+^ luminal tumors, overexpression of these genes correlates with altered prognosis and genomic instability. Annotation analysis further reveals a strong association between CNV patterns and cell proliferation rates in early-stage tumors (21, 89, 90). This link is further supported by the overlap between cell cycle gene sets and proliferation signatures in tumor samples (91). Notably, the expression of cell cycle effectors and regulators increases in concert with tumor cell proliferation rates. These genes, periodically expressed during the cell cycle, play critical roles in DNA replication, chromosome segregation, and spindle assembly (59, 92).

Recent evidence suggests that CCAs^21^, unlike NCCAs^22^, are the primary drivers of genomic instability and karyotype changes in early-stage tumors (93). Single-cell sequencing studies reveal that mammary epithelial cells in pre-cancerous lesions undergo numerous structural chromosomal aberrations, most of which are transient. Only a limited number of these alterations confer a selective advantage and are subjected to clonal selection (94, 95). The selection of initial clones with stable CNVs during tumor progression, even in the absence of proliferation gene modifications, can be explained by the immortalization model (86, 96).

The initial genomic variations in breast cancer, primarily consisting of stable CNVs, occur early in tumor progression and remain prevalent. In luminal subtype tumors, clonal evolution follows a punctuated pattern, with sub-branches emerging during tumor development, mainly through mutations and rarely through additional CNVs. In luminal B and a small subset of luminal A tumors, these new clones harbor oncogenes, altering the pattern of tumor evolution. This linear evolution leads to the accumulation of tumorigenic mutations in a single or limited number of sub-clones with proliferation support. In non-proliferative luminal A tumors, neutral evolution may occur, resulting in numerous sub-clones with no proliferation advantage (97, 98). However, when the initial clone lacks sufficient growth stimuli and selection occurs via a hallmark other than proliferation, such as immortalization, proliferation deficiency does not guarantee that mutated cancer cells in the clone will reach a significant population (96). Consequently, these tumors, with a low tumor mutation burden, consist of a large, slowly growing single clone that forms the bulk of the tumor mass, accompanied by numerous sub-clones with minimal abundance (98).

CNVs exert their phenotypic effects on tumors by encompassing long chromosome segments harboring multiple genes susceptible to amplifications and deletions. One crucial phenotypic property influenced by CNVs is proliferation, which directly impacts prognosis and therapeutic strategies, particularly in luminal breast tumors (99). However, neither Ki67 detection (a proliferation marker) nor histological findings provide reliable indicators of tumor proliferation propensity. The chromosomal instability index, exhibiting a strong correlation with CNV profiles in luminal breast tumors, offers a more accurate measure for predicting tumor progression compared to other markers (100).

(86, 93–96) Array-CGH^23^ results offer a promising approach for separating luminal subtypes based on their distinct chromosomal structural and numerical instability profiles. High-resolution CNV assays have revealed that specific CNVs are independently associated with low proliferation rates, low genomic instability, and favorable prognosis (101). These CNVs include gains of 1q and 16p and a loss of 16q, typically observed in luminal A tumors according to IHC^24^ criteria. Conversely, another group characterized by 8q loss and gains of 11q, 20q, and 17q is associated with high proliferation, intermediate-to-high genomic instability, and poor patient prognosis (86).

The Integrative Clustering (IntClust) approach, based on transcriptome data and prognosis, categorizes breast cancer tumors into 10 distinct groups (Table 5). Each IntClust exhibits a specific CNV pattern, intermediate/high genomic instability, and intermediate/poor 10-year prognosis. IntClust-1 and IntClust-5 largely correspond to conventional luminal B tumors and are closely related to the HER2 subtype due to 17q gain, which encompasses the ERBB2 (HER2) gene. IntClust-6 and IntClust-9 exhibit intermediate prognosis with variable IHC results. IntClust-6 is characterized by FGFR1 (Fibroblast Growth Factor Receptor 1) oncogene amplification on 8p, while IntClust-9 is defined by gains of 8q and 20q, leading to Myc and BHLH Transcription Factor overexpression, respectively. IntClust-2, associated with CCND1 (Cyclin D1) gene amplification on chromosome 11q, displays high genomic instability and a poor 10-year survival rate. IntClusts 3, 4, 7, and 8 primarily correspond to luminal A tumors with low to intermediate genomic instability and favorable 10-year survival. IntClust 3 and 4 exhibit minimal CNVs, while IntClust 7 and 8 subtypes display gains of 1q and 16p and a loss of 16q. Notably, 8q amplification, encompassing the Myc gene, is prevalent in IntClust7 but does not affect genomic instability, likely due to expression dosage insufficiency (7, 20, 59, 102, 103). Recent studies have confirmed that each CNV has a unique phenotypic impact. Chromosome 8q amplification, encompassing the Myc oncogene, is prevalent in ER^+^ breast tumors but has minimal effects on proliferation in many cases. This may be attributed to interactions between distant copy number variants and gene dosage dependencies (18, 61, 99). Table 5 provides an example of an integrated classification of breast cancer.

**TABLE 5:** Integrative Clustering (IntClust) of Breast tumors Variants.

IntClust	Frequency (%)	Profile of METABRIC integrative cluster	Chromosomal region and alteration type	HER2 status	PAM50 (%)	Clinical features	Prognosis	Genomic Instability	First-Line therapeutic decision	Ref.
1	67 (7.7%)	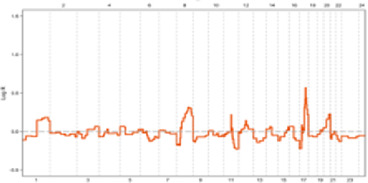	17q12 amp 20q13.2 amp 1q21-q44 amp 8p21-p23 amp GATA3 mut. (25%)	HER2+‏: 12.5% HER2-‏: 87.5%	LumA: 7.8% LumB: 75%	High grade	Intermediate (LPP)	High	CHT + HT	(45, 121, 145)
2	35 (2%)	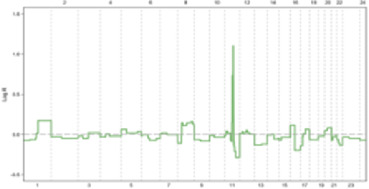	11q13 amp PIK3CA mut. (37.1%)	HER2+‏: 2.9% HER2-‏: 97,1%	LumA: 7.8% LumB: 75%	No distinct clinical features	Poor (LPP)	High	CHT + HT	
3	124 (14.9%)	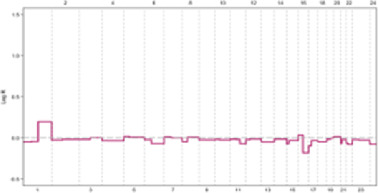	CNV devoid PIK3CA mut. (70.2%)	HER2+‏: 0% HER2-‏: 100%	LumA: 12.9% LumB: 71%	Low grade	Good (LGP)	Low	HT	
4	109 (13.1%)	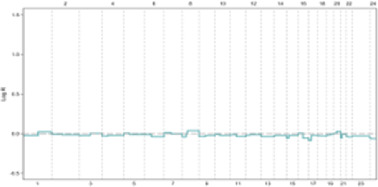	CNV devoid PIK3CA mut. (42.2%)	HER2+‏: 1.8% HER2-‏: 98.2%	LumA: 42.2% LumB: 13.8%	Low grade	Good (LGP)	Low	HT	
5	44 (5.3%)	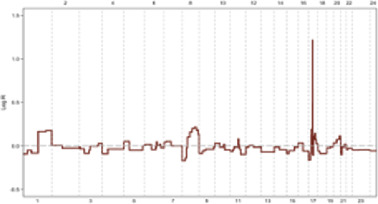	17q12 amp 17p13.1 amp	HER2+‏: 97.7% HER2-‏: 2.3%	LumA: 20.5% LumB: 50%	Younger age at diagnosis, High grade High LN‏+	Poor	high	CHT + HT	
6	41 (4.9%)	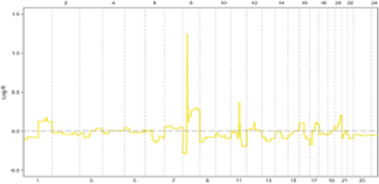	8p21-p23 del TP53 mut. (43.9%)	HER2+‏: 4.9% HER2-‏: 95.1%	LumA: 31.7% LumB: 46.3%	No distinct clinical features	Intermediate (LPP)	High	CHT + HT	
7	107 (12.8%)	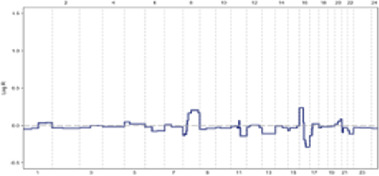	16q22.1 del PIK3CA mut. (51%)	HER2+‏: 0% HER2-‏: 100%	LumA: 67.3% LumB: 18.7%	Older age at diagnosis Low grade	Good (LGP)	Intermediate	HT	
8	133 (15.9%)	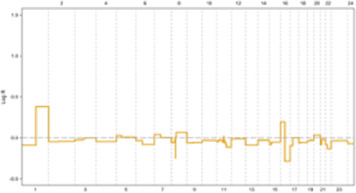	1q21-q44 amp 16q22.1 del PIK3CA mut. (54.9%)	HER2+‏: 0% HER2-‏: 100%	LumA: 71.4% LumB: 36.3%	Older age at diagnosis, Low grade	Good (LGP)	Intermediate	HT	
9	62 (7.4%)	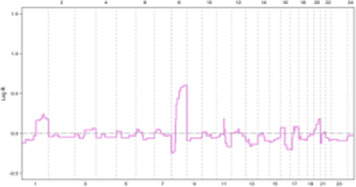	8q24 amp 8p21-p23 del 20q13.2 amp MYC amp. mut. (35.5%)	HER2+‏: 8.1% HER2-‏: 91.1%	LumA: 30.6% LumB: 58%	High grade	Intermediate (LPP)	High	CHT + HT	
10	16 (1.9%)	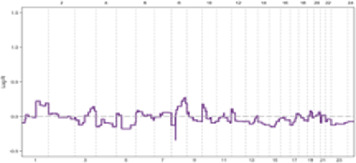	12p13.33 amp 8p21-p23 del 8q24 amp TP53 mut. (56.3%)	HER2+‏: 0% HER2-‏: 100%	LumA: 6.3% LumB: 68.8%	Younger age at diagnosis, High grade Large tumors	Poor	Intermediate	CHT + HT	

CHT: Chemo-hormone therapy, CT: chemotherapy, HT: hormone therapy, ER: estrogen receptor, HER2: Human Epidermal growth factor Receptor 2, (results method: IHC (Immunohistochemistry staining)), amp: amplification, de: deletion, mut. (red): mutation independent of target genetic variations. LPP: luminal poor prognosis, LGP: luminal good prognosis, Number of research patients: 1199, (Stage: 1&2 primary tumors). GATA3: GATA-binding protein 3, CCND1: cyclin D1, PIK3CA: Phosphatidylinositol-4, 5-Bisphosphate 3-Kinase Catalytic Subunit Alpha, LN: lymph node, FGFR1: Fibroblast Growth Factor Receptor 1, TP53: Tumor Protein P53, MYC: myelocytoma, a cancer of the myelocytes).

Immunostaining of 145 paraffin-embedded luminal breast cancer samples further supports the association between cell cycle activity and patient prognosis. Antibodies against MCM2 (Mini-chromosome Maintenance Complex Component 2), GMNN (Geminin DNA Replication Inhibitor, also known as “geminin”), AURKA (Aurora Kinase A), and Plk1 (Polo-like kinase 1) – all markers of cell cycle progression–were used for staining. Notably, 21% of non-stained ER^+^ samples were confirmed to be quiescent (G0 phase), 30% exhibited MCM2 staining alone indicating a prolonged G1 phase and 49% were stained by all antibodies, suggesting active progression through S, G2, and M phases. These findings demonstrate that approximately half of luminal breast cancer cells exhibit altered cell cycle activity, while the remaining half of mammary epithelial cells retain intrinsic sensitivity to mitogenic signaling. Consequently, tumors with an activated cell cycle may benefit from the use of phase-specific chemotherapeutic agents (Figure 3) (104, 105).

## 6 Discussion

Tumors with high *CDK2* transcripts were more likely to have higher expressions of genes involved in the cell cycle, homologous recombination, and p53 signaling. Genes/pathways involved in BC cell survival and proliferation were associated with worse outcomes, as opposed to most immune-related genes/signatures, especially in the CDK4/6i arm. CD24 was the only gene significantly associated with worse PFS in both arms. Tertiary lymphoid structures and higher tumor-infiltrating lymphocytes also showed favorable survival trends in the CDK4/6i arm (106).

The cell cycle, characterized by its invariant phase durations and independence between phases, normally proceeds through a tightly regulated sequence of molecular interactions, culminating in the error-free duplication of a mother cell into two daughter cells. The timing and impact of each molecular reaction are intricately linked to the cell’s genomic structure and epigenetic factors. Checkpoints within each phase play a critical role in ensuring the fidelity of cell cycle progression (99, 107, 108).

Luminal-type breast tumors are classified into Luminal A (ER^+^ and/or PR^+^ and HER2^-^) and Luminal B (ER^+^ and/or PR^+^ and HER2^+^) subtypes based on immunohistochemical staining. Tumor cells exhibit varying degrees of genomic alterations, which can perturb cell cycle progression. Disruption of checkpoint regulation in response to replication stress and DNA damage can lead to genomic instability (33, 56). This instability increases the propensity for cells to acquire new mutations and alter tumor evolution patterns, potentially affecting prognosis and treatment success.

Proper checkpoint activation triggers cell cycle arrest and activates DNA repair mechanisms to maintain genomic integrity and ensure the inheritance of a complete genome by daughter cells. If cellular compensatory mechanisms fail to repair the damage, prolonged arrest can initiate apoptosis (109). In cancer, the normally uncoupled nature of cell cycle phases becomes disrupted (110). This disruption can be driven by enhanced cross-talk signaling or the activity of transcription factors like E2F. Identifying these molecular players is crucial for selecting optimal therapies for each cancer type (111).

In the treatment of luminal breast cancer, the efficacy of CDK inhibitors in Luminal A and Topoisomerase II (Top II) inhibitors in Luminal B subtypes remains under investigation in clinical trials. Accurately predicting cell cycle alterations is crucial for selecting patient groups who will benefit from these therapies. This goal is achievable through the detection of copy number variations in early-stage tumors, leveraging the distinct genetic profiles of Luminal A and B subtypes (110). This approach holds promise for personalized treatment strategies, tailoring therapy to the specific genomic landscape of each patient’s tumor.

The recent FDA approval of CDK inhibitors, such as Palbociclib, has revolutionized the treatment landscape for HR^+^ metastatic breast cancer. These treatments have proven to be highly effective in improving patient outcomes when used in combination with tamoxifen, aromatase inhibitors, or Fulvestrant. This therapeutic regimen has also shown promise in hormone-receptor-positive, HER2-negative early-stage breast cancer, highlighting its potential for broader application (112).

Tailoring personalized medicine for breast cancer types Luminal A and Luminal B use their unique traits, particularly how they control the G1/S cell cycle step. Luminal A cancers are ER-positive (ER^+^), PR-positive (PR^+^), and HER2-negative (HER2^-^), with slow growth rates. Luminal B cancers, which are also ER^+^, tend to have lower PR levels, grow faster and may show HER2.

The G1/S checkpoint plays a key role in cell cycle control. Estrogen receptor signals boost cyclin D levels, which activates the CDK4/6-cyclin D complex. The activated complex adds phosphate to the Rb protein. Phosphorylation frees E2F transcription factors, allowing the cell cycle to move forward.

In Luminal A tumors, cell cycle regulatory systems like active p16INK4a and balanced CDK4/6 activity keep the G1/S step in arrest, leading to a slower growth phenomenon. Luminal B tumors, in contrast, often have issues displaying excessive CDK4/6 levels or cyclin D overexpression, loss of Rb, or p53 gene mutations, which cause fast uncontrolled proliferation.

Focusing on the CDK4/6-cyclin D is a possible efficient strategy. Palbociclib, a CDK4/6 inhibitor, stops the cell cycle in G1 by blocking Rb phosphorylated activation, sequestering E2F, and stopping growth. Studies like PALOMA-2 showed great gains in progression-free survival when Palbociclib was applied with letrozole for ER + breast cancers (113). For Luminal A, CDK4/6 inhibitors enhance hormone therapy efficiency, reducing recurrence/relapse events while displaying low toxicity. In Luminal B, these medications reduce the proliferation rate and synergize with other treatments like anti-HER2 drugs. This personalized guide, supported by molecular profiling data, optimizes treatment efficacy and significantly advances breast cancer medication management (102) DOI: 10.1016/j.molonc.2010.12.005).

The prognostic value of intrinsic subtypes in metastatic breast cancer suggests that tailoring chemotherapy regimens based on these subtypes may lead to improved outcomes (114, 115). While early-stage genetic profiles are generally considered invariant during metastasis, recent evidence suggests that tumor cells can acquire an E2F: OFF state through CDK4/6 inhibition (56). Notably, primary luminal A tumors with overexpression of mitogenic genes and CCND1, but not CCNE2, exhibit particular sensitivity to Palbociclib (56). This sensitivity is attributed to an indirect increase in P21 levels induced by Palbociclib, leading to cell cycle arrest in the G1 phase and accumulation of the G0 population within the tumor mass (116).

Recent studies suggest that the combination of limited oncogene activity and an intact spindle assembly checkpoint (SAC) function leads to genomic stability and a favorable prognosis. Conversely, perturbations in the cell cycle caused by overexpressed oncogenes and stable aneuploidy, coupled with a defective SAC function, induce genomic instability and a poor prognosis. The prevalence of stable aneuploidy and intact SAC machinery guides the therapeutic efficacy of paclitaxel and anthracyclines, a group of chemotherapeutic agents (110).

The prevalence of aneuploidy in Luminal B and a subset of Luminal A tumors suggests that these subtypes should be further categorized into two subgroups: diploid/CIN^−^ and aneuploid/CIN^+^, based on copy number variation patterns (77). While recent studies have indicated that SAC function may not be a significant factor in tumor progression, its unique machinery makes it a potential target for anti-cancer therapies. Microtubule-targeting agents (MTAs) like paclitaxel, currently used in metastatic breast cancer, have limited efficacy in Luminal B therapy due to the high aneuploidy and proliferation rate of these tumors (75).

Prolonged SAC inactivation forces cells to choose between two fates: initiating an SAC-originated apoptotic signal or progressing through the cell cycle with significant chromosome aneuploidy, exceeding the cellular tolerance for CINs (84). However, the impact of CNV patterns in cancer clinic utilization and the relationship between CNVs and the degree of genomic instability is not as straightforward in context. Recent studies indicate that in luminal breast tumors, the status of G1/S could control the proliferation rate of tumors due to the predictable E2F function in the presence of oncogenes overexpression, leading to a low genomic instability and a good prognosis. An identical CNV type leads to the E2F status change that acts as a critical transcription factor in G1 transition and S phase initiation. On the other hand, the status of the M phase checkpoint is highly dependent on CDK1/CycB function in APC/C-CDC20 activation. Cyclin B transcription is regulated by multiple transcription factors that only FoxM1 induced by CDK1/CycB among them, and others activated by various pathways. FoxM1 transcription factor that is activated by CDK1/CycB is one of the multiple transcription factors that lead to Cyclin B transcription. These findings suggest a linkage between the maintenance of aneuploidy/CIN and non-tumorigenic events in primary luminal breast tumors. These are cases where genomic instability results are biased from those predicted by CNV results (83, 117, 118).

However, the clinical implications of CNV patterns and their relationship to genomic instability remain complex. Recent studies in luminal breast tumors suggest that G1/S status may control tumor proliferation due to the predictable E2F function in the presence of oncogene overexpression, leading to low genomic instability and a favorable prognosis. Notably, an identical CNV type can induce changes in E2F status, which acts as a critical transcription factor in G1 transition and S phase initiation.

On the other hand, the M phase checkpoint status is tightly linked to CDK1/CycB function, which is essential for APC/C-CDC20 activation. A complex network of transcription factors, including FoxM1, which is specifically induced by CDK1/CycB, regulates cyclin B transcription. These findings suggest a potential link between the maintenance of aneuploidy/CIN and non-tumorigenic events in primary luminal breast tumors, where genomic instability results may deviate from those predicted by CNV analysis (83, 118).

### 6.1 Challenges

The copy of cell cycle genes can be changed directly by CNVs, which leads to their under-expression or overexpression. However, the exact mechanism that CNVs are involved in cell cycle control is unknown. To create specific treatments, it is important to distinguish between those CNVs that drive cell cycle progression and those that do not. Driver CNVs can be identified through functional studies and inter-tumor analyses. Luminal tumors display major differences in CNV profiles of different sub-clones. Single-cell sequencing is necessary to assess how CNVs affect cellular cycle dynamics and identify their clones’ evolution patterns. CNVs are frequently observed together with other genetic alterations, and to address treatment strategies for cell cycle malfunction, it is important to comprehend their interaction. Different technologies known as multi-omics are used to generate data that show an overview of the molecular signature and influence on the cell cycle’s regulation. Even though the CNVs bring hope to picking patients responsive to cell cycle deregulation-targeted treatments, the implementation of these discoveries in a medical environment is still difficult. Knowledge of the links between CNVs and cell cycle checkpoints can give a foundation for individualized medication approaches. Enabling patient-specific therapeutic interventions with specific CNV patterns and state of checkpoints would enhance treatment efficiency and patient outcomes. It is necessary to make sure that strong models predicting future events simultaneously help in realizing biomarkers linked to CNV-induced cell-cycle changes on an individualized basis. To successfully face these challenges, it is necessary to use collaborative methods of working and using genomics, cell biology, and clinical oncology knowledge. Understanding how CNVs interact with aberrations in cell cycle regulation is pivotal to the development of better luminal-type breast cancer treatment strategies, which are personalized as well.

## 7 Conclusion

The effect of CNVs on cell cycle dysregulation in luminal-type breast cancer has been thoroughly discussed in this study. We have discussed how CNVs can cause genomic instability and tumor growth by altering cell cycle checkpoints when they occur alone or in conjunction with other variables. Results point to a critical role for CNVs in the initiation and spread of luminal-type breast cancer. By identifying how CNVs disrupt cell cycle checkpoints, the findings offer a foundational framework for developing targeted therapeutic strategies. These findings have given the basic framework to develop targeted therapeutic strategies by pointing out how CNVs disrupt cell cycle checkpoints. This may integrate CNV profiling into clinical practice and allow patient stratification based on genomic alterations to open the door to precision oncology interventions. Because of the genes targeted by CNVs, there is indeed a prospect to build very personal therapeutic regimens. Examples are CNV correction, leveraging CRISPR-Cas9 technology against the aberrant copies induced, or the application of inhibitors that may be developed for dysregulated pathways within the cell cycle. New findings point toward an exceptional ability of biomarker-driven therapies in general, a successful experience well noted in the HER2 and CDK4/6 pathway treatments. Future studies should be focused on high-throughput genomic analyses and integrative multi-omics approaches to identify actionable targets affected by CNVs. Thus, the correlation of CNVs with therapeutic responses will advance the development of novel treatment modalities in concert with individual patient profiles and improve outcomes in this aggressive subtype of breast cancer.

## Footnotes

1 Spindle Assembly Checkpoint.

2 Cyclin-Dependent Kinases.

3 Retinoblastoma Protein.

4 Origin Recognition Complex.

5 Ataxia Telangiectasia Mutated.

6 Ataxia Telangiectasia and Rad3-Related Protein.

7 DNA-Dependent Protein Kinase Catalytic Subunit.

8 Double-Strand Breaks.

9 Single-Strand Breaks.

10 Checkpoint Kinase 1.

11 Transcription-Replication Conflicts.

12 Forkhead Box M1.

13 BORA Aurora Kinase A Activator.

14 Checkpoint Kinase 1&2.

15 Common Fragile Sites.

16 Homologous Recombination.

17 Tumor Protein p53.

18 Anaphase-Promoting Complex/Cyclosome.

19 Cyclosome-Cell Division Cycle 20.

20 Numerical Chromosome Instability.

21 Clonal Chromosomal Aberrations.

22 Non-Clonal Aberrations.

23 Array Comparative Genomic Hybridization.

24 Immunohistochemistry.

## References

[1] Takeshima H Tjnpo U. Accumulation of genetic and epigenetic alterations in normal cells and cancer risk. (2019) 3(1):1–8. 10.1038/s41698-019-0079-0 PMC6403339 30854468

[2] Pal B Chen Y Vaillant F Capaldo BD Joyce R Song X A single‐cell RNA expression atlas of normal, preneoplastic and tumorigenic states in the human breast. The EMBO J (2021) 40(11):e107333. 10.15252/embj.2020107333 33950524 PMC8167363

[3] Siegel RL Miller KD Fuchs HE Jemal A. Cancer statistics. CA: a Cancer J clinicians (2022) 72(1):7–33. 10.3322/caac.21708 35020204

[4] Hashemi F Mohajeri N Radnia F Zarghami N. Design of an efficient fluorescent nanoplatform carrier for hydrophobic drugs along with green carbon dot: possible application in cancer image-guided drug therapy. Photodiagnosis Photodynamic Ther (2022) 37:102738. 10.1016/j.pdpdt.2022.102738 35074467

[5] Blows FM Driver KE Schmidt MK Broeks A Van Leeuwen FE Wesseling J Subtyping of breast cancer by immunohistochemistry to investigate a relationship between subtype and short and long term survival: a collaborative analysis of data for 10,159 cases from 12 studies. PLoS Med (2010) 7(5):e1000279. 10.1371/journal.pmed.1000279 20520800 PMC2876119

[6] Perou CM Sørlie T Eisen MB Van De Rijn M Jeffrey SS Rees CA Molecular portraits of human breast tumours. nature (2000) 406(6797):747–52. 10.1038/35021093 10963602

[7] Dawson SJ Rueda OM Aparicio S Caldas C. A new genome‐driven integrated classification of breast cancer and its implications. The EMBO J (2013) 32(5):617–28. 10.1038/emboj.2013.19 23395906 PMC3590990

[8] Turk Z Armani A Jafari-Gharabaghlou D Madakbas S Bonabi E Zarghami N. A new insight into the early detection of HER2 protein in breast cancer patients with a focus on electrochemical biosensors approaches: a review. Int J Biol Macromolecules (2024) 272:132710. 10.1016/j.ijbiomac.2024.132710 38825266

[9] Di Palma S Koliou P Simonovic A Costa D Faulkes C Kobutungi B Breast cancer molecular subtyping in practice: a real-world study of the APIS breast cancer subtyping assay in a consecutive series of breast core biopsies. Int J Mol Sci (2024) 25(5):2616. 10.3390/ijms25052616 38473863 PMC10931915

[10] Sarhadi S Armani A Jafari-Gharabaghlou D Sadeghi S Zarghami N. Cross-platform gene expression profiling of breast cancer: exploring the relationship between breast cancer grades and gene expression pattern. Heliyon (2024) 10(8):e29736. 10.1016/j.heliyon.2024.e29736 38681607 PMC11053269

[11] Özmen V Özmen T Doğru V. Breast cancer in Turkey; an analysis of 20.000 patients with breast cancer. Eur J Breast Health (2019) 15(3):141–6. 10.5152/ejbh.2019.4890 31312788 PMC6619786

[12] Ciriello G Sinha R Hoadley KA Jacobsen AS Reva B Perou CM The molecular diversity of Luminal A breast tumors. Breast Cancer Res Treat (2013) 141(3):409–20. 10.1007/s10549-013-2699-3 24096568 PMC3824397

[13] Mohammadinejad S Jafari-Gharabaghlou D Zarghami N. Development of PEGylated PLGA nanoparticles co-loaded with bioactive compounds: potential anticancer effect on breast cancer cell lines. Asian Pac J Cancer Prev (2022) 23(12):4063–72. 10.31557/apjcp.2022.23.12.4063 36579986 PMC9971482

[14] Negrini S Gorgoulis VG Halazonetis TD. Genomic instability—an evolving hallmark of cancer. Nat Rev Mol Cel Biol (2010) 11(3):220–8. 10.1038/nrm2858 20177397

[15] Duijf PH Nanayakkara D Nones K Srihari S Kalimutho M Khanna KK. Mechanisms of genomic instability in breast cancer. Trends Molecular Medicine (2019) 25(7):595–611. 10.1016/j.molmed.2019.04.004 31078431

[16] Colnaghi R Carpenter G Volker M O’Driscoll M, editors. The consequences of structural genomic alterations in humans: genomic disorders, genomic instability and cancer. Seminars in cell and developmental biology. Elsevier (2011). 10.1016/j.semcdb.2011.07.010 21802523

[17] Zhang F Gu W Hurles ME Lupski JR. Copy number variation in human health, disease, and evolution. Annu Rev genomics Hum Genet (2009) 10:451–81. 10.1146/annurev.genom.9.081307.164217 19715442 PMC4472309

[18] Gatza ML Silva GO Parker JS Fan C Perou CM. An integrated genomics approach identifies drivers of proliferation in luminal-subtype human breast cancer. Nat Genet (2014) 46(10):1051–9. 10.1038/ng.3073 25151356 PMC4300117

[19] Bergamaschi A Kim YH Wang P Sørlie T Hernandez‐Boussard T Lonning PE Distinct patterns of DNA copy number alteration are associated with different clinicopathological features and gene‐expression subtypes of breast cancer. Genes, Chromosomes and Cancer (2006) 45(11):1033–40. 10.1002/gcc.20366 16897746

[20] Ali HR Rueda OM Chin S-F Curtis C Dunning MJ Aparicio SA Genome-driven integrated classification of breast cancer validated in over 7,500 samples. Genome Biol (2014) 15(8):431–14. 10.1186/s13059-014-0431-1 25164602 PMC4166472

[21] Tishchenko I Milioli HH Riveros C Moscato P. Extensive transcriptomic and genomic analysis provides new insights about luminal breast cancers. PloS one (2016) 11(6):e0158259. 10.1371/journal.pone.0158259 27341628 PMC4920434

[22] Shahrouzi P Forouz F Mathelier A Kristensen VN Duijf PHG. Copy number alterations: a catastrophic orchestration of the breast cancer genome. Trends Mol Med (2024) 30(8):750–64. 10.1016/j.molmed.2024.04.017 38772764

[23] Wang Y-W Tuan Y-L Wang J-Y Chang H-Y Chu C-A Chen Y-L Potential of epithelial membrane protein 3 as a novel therapeutic target for human breast cancer. Oncol Rep (2024) 53(1):16–3. 10.3892/or.2024.8849 39611484 PMC11632653

[24] Zhang Y Xu H Han X Yu Q Zheng L Xiao H. PMAIP1-mediated glucose metabolism and its impact on the tumor microenvironment in breast cancer: integration of multi-omics analysis and experimental validation. Translational Oncol (2025) 52:102267. 10.1016/j.tranon.2024.102267 PMC11750568 39740516

[25] Koon Sun Pat M Manraj M Manraj S. Breast cancer survival analysis in the Republic of Mauritius by age, stage at diagnosis and molecular subtype: a retrospective cohort study. Int J Cancer (2025) 156(2):331–8. 10.1002/ijc.35172 39243396

[26] Yu X Zhang Q Li B Sun S Li J Li W. Unveiling the prognostic power and immune landscape of MyD88 in breast cancer: an integrative bioinformatics and IHC approach. J Cancer (2025) 16(1):201–13. 10.7150/jca.103403 39744584 PMC11660130

[27] Pan X Hu X Zhang Y-H Chen L Zhu L Wan S Identification of the copy number variant biomarkers for breast cancer subtypes. Mol Genet Genomics (2019) 294:95–110. 10.1007/s00438-018-1488-4 30203254

[28] Mekonnen N Yang H Rajasekaran N Song K Choi Y-L Shin YK. Indirect targeting of MYC and direct targeting in combination with chemotherapies are more effective than direct mono-targeting in triple negative breast cancer. Translational Oncol (2025) 51:102204. 10.1016/j.tranon.2024.102204 PMC11652953 39631207

[29] Dall'Olio FG Zrafi W Mamann A Job B Michiels S Tomasini P Genomic instability in advanced non-small cell lung cancer (NSCLC) treated with maintenance durvalumab in the UNICANCER SAFIR02-Lung/IFCT1301 trial. American Society of Clinical Oncology (2024).

[30] Li W Wu H Xu J. Construction of a genomic instability-derived predictive prognostic signature for non-small cell lung cancer patients. Cancer Genet (2023) 278-279:24–37. 10.1016/j.cancergen.2023.07.008 37579716

[31] Liu Y Bi X Leng Y Chen D Wang J Ma Y A deep-learning-based genomic status estimating framework for homologous recombination deficiency detection from low-pass whole genome sequencing. Heliyon (2024) 10:e26121. 10.1016/j.heliyon.2024.e26121 38404843 PMC10884843

[32] Sahabi S Jafari-Gharabaghlou D Zarghami N. A new insight into cell biological and biochemical changes through aging. Acta Histochem (2022) 124(1):151841. 10.1016/j.acthis.2021.151841 34995929

[33] Wilhelm T Said M Naim V. DNA replication stress and chromosomal instability: dangerous liaisons. Genes (2020) 11(6):642. 10.3390/genes11060642 32532049 PMC7348713

[34] Sayad S Hiatt M Mustafa H. Connecting cancers to chromosomal locations through copy number variations. bioRxiv. (2023). 10.1101/2023.10.03.560705

[35] Pool EJ Walters CR Pool EJ. The effects of silver nanoparticles on RAW 264.7. Macrophages and human whole blood cell cultures. Front Biosci (2019) 24(2):347–65. 10.2741/4722 30468660

[36] Luo X Qin F Cai G Xiao F. Integrating genomic correlation structure improves copy number variations detection. Bioinformatics (2021) 37(3):312–7. 10.1093/bioinformatics/btaa737 32805016

[37] Macé A Kutalik Z Valsesia A. Copy number variation. In: Evangelou E, editor. Genetic epidemiology: methods and protocols. New York, NY: Springer New York (2018). p. 231–58. 10.1007/978-1-4939-7868-7_14 29876900

[38] Huang T. Copy number variations in tumors. (2019).

[39] Leclerc S Kitagawa K. The role of human centromeric RNA in chromosome stability. Front Mol Biosciences (2021) 8:642732. 10.3389/fmolb.2021.642732 PMC8044762 33869284

[40] Camargo-Herrera V Castellanos G Rangel N Jiménez-Tobón GA Martínez-Agüero M Rondón-Lagos M. Patterns of chromosomal instability and clonal heterogeneity in luminal B breast cancer: a pilot study. Int J Mol Sci (2024) 25(8):4478. 10.3390/ijms25084478 38674062 PMC11049937

[41] Voutsadakis IA. The landscape of chromosome instability in breast cancers and associations with the tumor mutation burden: an analysis of data from TCGA. Cancer Invest (2021) 39(1):25–38. 10.1080/07357907.2020.1863418 33306412

[42] Lee K Kim HJ Jang MH Lee S Ahn S Park SY. Centromere 17 copy number gain reflects chromosomal instability in breast cancer. Scientific Rep (2019) 9(1):17968. 10.1038/s41598-019-54471-w PMC6884473 31784614

[43] Brewer T Yehia L Bazeley P Eng C. Integrating somatic CNV and gene expression in breast cancers from women with PTEN hamartoma tumor syndrome. NPJ Genomic Med (2023) 8(1):14. 10.1038/s41525-023-00361-0 PMC10322985 37407629

[44] Gabrielaite M Torp MH Rasmussen MS Andreu-Sánchez S Vieira FG Pedersen CB A comparison of tools for copy-number variation detection in germline whole exome and whole genome sequencing data. Cancers (Basel) (2021) 13(24):6283. 10.3390/cancers13246283 34944901 PMC8699073

[45] Young CC Eason K Manzano Garcia R Moulange R Mukherjee S Chin SF Development and validation of a reliable DNA copy-number-based machine learning algorithm (CopyClust) for breast cancer integrative cluster classification. Sci Rep (2024) 14(1):11861. 10.1038/s41598-024-62724-6 38789621 PMC11126405

[46] Savarese F Grosschedl R. Blurring cis and trans in gene regulation. Cell (2006) 126(2):248–50. 10.1016/j.cell.2006.07.008 16873057

[47] Kanu N Grönroos E Martinez P Burrell RA Yi Goh X Bartkova J SETD2 loss-of-function promotes renal cancer branched evolution through replication stress and impaired DNA repair. Oncogene (2015) 34(46):5699–708. 10.1038/onc.2015.24 25728682 PMC4660036

[48] Cavalli FMG Remke M Rampasek L Peacock J Shih DJH Luu B Intertumoral heterogeneity within medulloblastoma subgroups. Cancer Cell (2017) 31(6):737–54.e6. 10.1016/j.ccell.2017.05.005 28609654 PMC6163053

[49] Danforth DN Jr. Genomic changes in normal breast tissue in women at normal risk or at high risk for breast cancer. Breast Cancer Basic Clin Res (2016) 10:BCBCR.S39384–46. 10.4137/bcbcr.s39384 PMC4990153 27559297

[50] Morgan DO. The cell cycle: principles of control. Yale Journal of Biology and Medicine (YJBM) (2007).

[51] Fischer M Dang CV DeCaprio JA. Control of cell division. Hematology. Elsevier (2018). p. 176–85.

[52] Shehata M Waterhouse PD Casey AE Fang H Hazelwood L Khokha R. Proliferative heterogeneity of murine epithelial cells in the adult mammary gland. Commun Biol (2018) 1(1):111–0. 10.1038/s42003-018-0114-7 30271991 PMC6123670

[53] Kwon JS Everetts NJ Wang X Wang W Della Croce K Xing J Controlling depth of cellular quiescence by an Rb-E2F network switch. Cell Rep (2017) 20(13):3223–35. 10.1016/j.celrep.2017.09.007 28954237 PMC6571029

[54] Amirsaadat S Jafari-Gharabaghlou D Dadashpour M Zarghami N. Potential anti-proliferative effect of nano-formulated curcumin through modulating micro RNA-132, Cyclin D1, and hTERT genes expression in breast cancer cell lines. J Cluster Sci (2023) 34(5):2537–46. 10.1007/s10876-023-02404-z

[55] Barr AR Cooper S Heldt FS Butera F Stoy H Mansfeld J DNA damage during S-phase mediates the proliferation-quiescence decision in the subsequent G1 via p21 expression. Nat Commun (2017) 8(1):14728–17. 10.1038/ncomms14728 28317845 PMC5364389

[56] Matthews HK Bertoli C de Bruin RA. Cell cycle control in cancer. Nat Rev Mol Cell Biol (2022) 23(1):74–88. 10.1038/s41580-021-00404-3 34508254

[57] Lashen A Alqahtani S Shoqafi A Algethami M Jeyapalan JN Mongan NP Clinicopathological significance of cyclin-dependent kinase 2 (CDK2) in ductal carcinoma *in situ* and early-stage invasive breast cancers. Int J Mol Sci (2024) 25(9):5053. 10.3390/ijms25095053 38732271 PMC11084890

[58] O’Connor TN Schultz E Wang J O’Connor T Levine E Knudsen ES Real-world experience among elderly metastatic breast cancer patients treated with CDK4/6 inhibitor-based therapy. Cancers. (2024) 16(9):1749. 10.3390/cancers16091749 38730702 PMC11083425

[59] Whitfield ML George LK Grant GD Perou CM. Common markers of proliferation. Nat Rev Cancer (2006) 6(2):99–106. 10.1038/nrc1802 16491069

[60] Kent LN Leone G. The broken cycle: E2F dysfunction in cancer. Nat Rev Cancer (2019) 19(6):326–38. 10.1038/s41568-019-0143-7 31053804

[61] Baslan T Kendall J Volyanskyy K McNamara K Cox H D'Italia S Novel insights into breast cancer copy number genetic heterogeneity revealed by single-cell genome sequencing. elife (2020) 9:e51480. 10.7554/elife.51480 32401198 PMC7220379

[62] Tyson JJ Chen KC Novak B. Sniffers, buzzers, toggles and blinkers: dynamics of regulatory and signaling pathways in the cell. Curr Opin Cel Biol (2003) 15(2):221–31. 10.1016/s0955-0674(03)00017-6 12648679

[63] Stallaert W Kedziora KM Chao HX Purvis JE. Bistable switches as integrators and actuators during cell cycle progression. FEBS Lett (2019) 593(20):2805–16. 10.1002/1873-3468.13628 31566708 PMC7881439

[64] Rombouts J Gelens L. Dynamic bistable switches enhance robustness and accuracy of cell cycle transitions. PLoS Comput Biol (2021) 17(1):e1008231. 10.1371/journal.pcbi.1008231 33411761 PMC7817062

[65] Yao G Lee TJ Mori S Nevins JR You L. A bistable Rb–E2F switch underlies the restriction point. Nat Cel Biol (2008) 10(4):476–82. 10.1038/ncb1711 18364697

[66] Otto T Sicinski P. Cell cycle proteins as promising targets in cancer therapy. Nat Rev Cancer (2017) 17(2):93–115. 10.1038/nrc.2016.138 28127048 PMC5345933

[67] Gemble S Bernhard S Srivastava N Wardenaar R Nano M Macé A-S Mechanisms of genetic instability in a single S-phase following whole genome doubling. (2021).

[68] Martínez-Alonso D Malumbres M, editors. Mammalian cell cycle cyclins. Seminars in cell and developmental biology. Elsevier (2020). 10.1016/j.semcdb.2020.03.009 32334991

[69] Willems E Dedobbeleer M Digregorio M Lombard A Lumapat PN Rogister B. The functional diversity of Aurora kinases: a comprehensive review. Cell division (2018) 13(1):7–17. 10.1186/s13008-018-0040-6 30250494 PMC6146527

[70] Hartwell LH Kastan MB. Cell cycle control and cancer. Science (1994) 266(5192):1821–8. 10.1126/science.7997877 7997877

[71] Wilson TE Arlt MF Park SH Rajendran S Paulsen M Ljungman M Large transcription units unify copy number variants and common fragile sites arising under replication stress. Genome Res (2015) 25(2):189–200. 10.1101/gr.177121.114 25373142 PMC4315293

[72] Williams RM Zhang X. Roles of ATM and ATR in DNA double strand breaks and replication stress. Prog Biophys Mol Biol (2021) 161:27–38. 10.1016/j.pbiomolbio.2020.11.005 33259832

[73] Ozeri-Galai E Schwartz M Rahat A Kerem B. Interplay between ATM and ATR in the regulation of common fragile site stability. Oncogene (2008) 27(15):2109–17. 10.1038/sj.onc.1210849 17934520

[74] Vijayraghavan S Tsai F-L Schwacha A. A checkpoint-related function of the MCM replicative helicase is required to avert accumulation of RNA: DNA hybrids during S-phase and ensuing DSBs during G2/M. PLoS Genet (2016) 12(8):e1006277. 10.1371/journal.pgen.1006277 27556397 PMC4996524

[75] Marques S Fonseca J Silva P Bousbaa H. Targeting the spindle assembly checkpoint for breast cancer treatment. Curr Cancer Drug Targets (2015) 15(4):272–81. 10.2174/1568009615666150302130010 25731686

[76] Martínez-A C van Wely KH. Are aneuploidy and chromosome breakage caused by a CINgle mechanism? Cell Cycle (2010) 9(12):2275–80. 10.4161/cc.9.12.11865 20519949 PMC13048558

[77] Yanagawa M Ikemot K Kawauchi S Furuya T Yamamoto S Oka M Luminal A and luminal B (HER2 negative) subtypes of breast cancer consist of a mixture of tumors with different genotype. BMC Res Notes (2012) 5(1):376–8. 10.1186/1756-0500-5-376 22830453 PMC3413599

[78] Pfister K Pipka JL Chiang C Liu Y Clark RA Keller R Identification of drivers of aneuploidy in breast tumors. Cell Rep (2018) 23(9):2758–69. 10.1016/j.celrep.2018.04.102 29847804 PMC5997284

[79] Wenzel ES Singh AT. Cell-cycle checkpoints and aneuploidy on the path to cancer. In vivo (Athens, Greece) (2018) 32(1):1–5. 10.21873/invivo.11197 29275292 PMC5892633

[80] Faria J Barbosa J Moura IM Reis RM Bousbaa H. The spindle assembly checkpoint and aneuploidy. Aneuploidy: etiology. The New England Journal of Medicine (2012). 59–76. Disorders and Risk Factors.

[81] Barbosa J Nascimento AV Faria J Silva P Bousbaa H. The spindle assembly checkpoint: perspectives in tumorigenesis and cancer therapy. Front Biol (2011) 6(2):147–55. 10.1007/s11515-011-1122-x

[82] Lara-Gonzalez P Moyle MW Budrewicz J Mendoza-Lopez J Oegema K Desai A. The G2-to-M transition is ensured by a dual mechanism that protects cyclin B from degradation by Cdc20-activated APC/C. Developmental Cel (2019) 51(3):313–25. e10. 10.1016/j.devcel.2019.09.005 PMC7778526 31588029

[83] Fischer M Müller GA. Cell cycle transcription control: DREAM/MuvB and RB-E2F complexes. Crit Rev Biochem Mol Biol (2017) 52(6):638–62. 10.1080/10409238.2017.1360836 28799433

[84] Cohen-Sharir Y McFarland JM Abdusamad M Marquis C Bernhard SV Kazachkova M Aneuploidy renders cancer cells vulnerable to mitotic checkpoint inhibition. Nature (2021) 590(7846):486–91. 10.1038/s41586-020-03114-6 33505028 PMC8262644

[85] Ciriello G Miller ML Aksoy BA Senbabaoglu Y Schultz N Sander C. Emerging landscape of oncogenic signatures across human cancers. Nat Genet (2013) 45(10):1127–33. 10.1038/ng.2762 24071851 PMC4320046

[86] Rennstam K Ahlstedt-Soini M Baldetorp B Bendahl P-O Borg Å Karhu R Patterns of chromosomal imbalances defines subgroups of breast cancer with distinct clinical features and prognosis. A study of 305 tumors by comparative genomic hybridization. Cancer Res (2003) 63(24):8861–8. 14695203

[87] Arsuaga J Borrman T Cavalcante R Gonzalez G Park C. Identification of copy number aberrations in breast cancer subtypes using persistence topology. Microarrays (2015) 4(3):339–69. 10.3390/microarrays4030339 27600228 PMC4996377

[88] Cava C Pisati M Frasca M Castiglioni I. Identification of breast cancer subtype-specific biomarkers by integrating copy number alterations and gene expression profiles. Medicina (2021) 57(3):261. 10.3390/medicina57030261 33809336 PMC7998437

[89] Fumagalli C Ranghiero A Gandini S Corso F Taormina S De Camilli E Inter-tumor genomic heterogeneity of breast cancers: comprehensive genomic profile of primary early breast cancers and relapses. Breast Cancer Res (2020) 22(1):107–11. 10.1186/s13058-020-01345-z 33059724 PMC7566144

[90] Aure MR Vitelli V Jernström S Kumar S Krohn M Due EU Integrative clustering reveals a novel split in the luminal A subtype of breast cancer with impact on outcome. Breast Cancer Res (2017) 19(1):44–18. 10.1186/s13058-017-0812-y 28356166 PMC5372339

[91] Perou CM Jeffrey SS Van De Rijn M Rees CA Eisen MB Ross DT Distinctive gene expression patterns in human mammary epithelial cells and breast cancers. Proc Natl Acad Sci (1999) 96(16):9212–7. 10.1073/pnas.96.16.9212 10430922 PMC17759

[92] Rhodes DR Yu J Shanker K Deshpande N Varambally R Ghosh D Large-scale meta-analysis of cancer microarray data identifies common transcriptional profiles of neoplastic transformation and progression. Proc Natl Acad Sci (2004) 101(25):9309–14. 10.1073/pnas.0401994101 15184677 PMC438973

[93] Heng HH Stevens JB Liu G Bremer SW Ye KJ Reddy PV Stochastic cancer progression driven by non‐clonal chromosome aberrations. J Cell Physiol (2006) 208(2):461–72. 10.1002/jcp.20685 16688757

[94] Wang Y Waters J Leung ML Unruh A Roh W Shi X Clonal evolution in breast cancer revealed by single nucleus genome sequencing. Nature (2014) 512(7513):155–60. 10.1038/nature13600 25079324 PMC4158312

[95] Navin N Kendall J Troge J Andrews P Rodgers L McIndoo J Tumour evolution inferred by single-cell sequencing. Nature (2011) 472(7341):90–4. 10.1038/nature09807 21399628 PMC4504184

[96] Horne SD Wexler M Stevens JB Heng HH. Insights on processes of evolutionary tumor growth. In: Atlas of genetics and cytogenetics in oncology and haematology (2016).

[97] Poudel P Nyamundanda G Patil Y Cheang MCU Sadanandam A. Heterocellular gene signatures reveal luminal-A breast cancer heterogeneity and differential therapeutic responses. NPJ breast cancer (2019) 5(1):21–10. 10.1038/s41523-019-0116-8 31396557 PMC6677833

[98] Vendramin R Litchfield K Swanton C. Cancer evolution: Darwin and beyond. The EMBO J (2021) 40(18):e108389. 10.15252/embj.2021108389 34459009 PMC8441388

[99] Dexter TJ Sims D Mitsopoulos C Mackay A Grigoriadis A Ahmad AS Genomic distance entrained clustering and regression modelling highlights interacting genomic regions contributing to proliferation in breast cancer. BMC Syst Biol (2010) 4(1):127–14. 10.1186/1752-0509-4-127 20825665 PMC2946304

[100] Vincent-Salomon A Benhamo V Gravier E Rigaill G Gruel N Robin S Genomic instability: a stronger prognostic marker than proliferation for early stage luminal breast carcinomas. PLoS One (2013) 8(10):e76496. 10.1371/journal.pone.0076496 24143191 PMC3797106

[101] Bonnet F Guedj M Jones N Sfar S Brouste V Elarouci N An array CGH based genomic instability index (G2I) is predictive of clinical outcome in breast cancer and reveals a subset of tumors without lymph node involvement but with poor prognosis. BMC Med genomics (2012) 5(1):54–18. 10.1186/1755-8794-5-54 23186559 PMC3558323

[102] Curtis C Shah SP Chin S-F Turashvili G Rueda OM Dunning MJ The genomic and transcriptomic architecture of 2,000 breast tumours reveals novel subgroups. Nature (2012) 486(7403):346–52. 10.1038/nature10983 22522925 PMC3440846

[103] Imani M Mohajeri N Rastegar M Zarghami N. Synthesis and characterization of N-rich fluorescent bio-dots as a reporter in the design of dual-labeled FRET probe for TaqMan PCR: a feasibility study. Biotechnol Appl Biochem (2023) 70(2):645–58. 10.1002/bab.2387 35900086

[104] Loddo M Kingsbury S Rashid M Proctor I Holt C Young J Cell-cycle-phase progression analysis identifies unique phenotypes of major prognostic and predictive significance in breast cancer. Br J Cancer (2009) 100(6):959–70. 10.1038/sj.bjc.6604924 19240714 PMC2661794

[105] Shetty A Loddo M Fanshawe T Prevost A Sainsbury R Williams G DNA replication licensing and cell cycle kinetics of normal and neoplastic breast. Br J Cancer (2005) 93(11):1295–300. 10.1038/sj.bjc.6602829 16278669 PMC2361513

[106] Schettini F Palleschi M Mannozzi F Brasó-Maristany F Cecconetto L Galván P CDK4/6-Inhibitors versus chemotherapy in advanced HR+/HER2-Negative breast cancer: results and correlative biomarker analyses of the KENDO randomized phase II trial. The Oncologist (2024):oyad337. 10.1093/oncolo/oyad337 PMC11067809 38175669

[107] Chao HX Fakhreddin RI Shimerov HK Kedziora KM Kumar RJ Perez J Evidence that the human cell cycle is a series of uncoupled, memoryless phases. Mol Syst Biol (2019) 15(3):e8604. 10.15252/msb.20188604 30886052 PMC6423720

[108] Bueno MJ Malumbres M. MicroRNAs and the cell cycle. Biochim Biophys Acta (BBA)-Molecular Basis Dis (2011) 1812(5):592–601. 10.1016/j.bbadis.2011.02.002 21315819

[109] Chen J. The cell-cycle arrest and apoptotic functions of p53 in tumor initiation and progression. Cold Spring Harbor Perspect Med (2016) 6(3):a026104. 10.1101/cshperspect.a026104 PMC4772082 26931810

[110] Bower JJ Vance LD Psioda M Smith-Roe SL Simpson DA Ibrahim JG Patterns of cell cycle checkpoint deregulation associated with intrinsic molecular subtypes of human breast cancer cells. NPJ Breast Cancer (2017) 3(1):9–12. 10.1038/s41523-017-0009-7 28649649 PMC5445620

[111] Heldt FS Barr AR Cooper S Bakal C Novák B. A comprehensive model for the proliferation–quiescence decision in response to endogenous DNA damage in human cells. Proc Natl Acad Sci (2018) 115(10):2532–7. 10.1073/pnas.1715345115 29463760 PMC5877942

[112] Mayer EL Dueck AC Martin M Rubovszky G Burstein HJ Bellet-Ezquerra M Palbociclib with adjuvant endocrine therapy in early breast cancer (PALLAS): interim analysis of a multicentre, open-label, randomised, phase 3 study. The Lancet Oncol (2021) 22(2):212–22. 10.1016/s1470-2045(20)30642-2 33460574

[113] Finn RS Martin M Rugo HS Jones S Im SA Gelmon K Palbociclib and letrozole in advanced breast cancer. N Engl J Med (2016) 375(20):1925–36. 10.1056/nejmoa1607303 27959613

[114] Zardavas D Baselga J Piccart M. Emerging targeted agents in metastatic breast cancer. Nat Rev Clin Oncol (2013) 10(4):191–210. 10.1038/nrclinonc.2013.29 23459626

[115] Prat A Ellis MJ Perou CM. Practical implications of gene-expression-based assays for breast oncologists. Nat Rev Clin Oncol (2012) 9(1):48–57. 10.1038/nrclinonc.2011.178 PMC3703639 22143140

[116] Serra F Lapidari P Quaquarini E Tagliaferri B Sottotetti F Palumbo R. Palbociclib in metastatic breast cancer: current evidence and real-life data. Drugs in context. (2019) 8:1–16. 10.7573/dic.212579 PMC6668507 31391852

[117] Memari F Tavakolpour V Mohajeri N Poopak B Fallah P Alizadeh E Distinct power of bone marrow microRNA signatures and tumor suppressor genes for early detection of acute leukemia. Clin Transl Oncol (2022) 24(7):1372–80. 10.1007/s12094-022-02781-3 35247197

[118] Müller GA Wintsche A Stangner K Prohaska SJ Stadler PF Engeland K. The CHR site: definition and genome-wide identification of a cell cycle transcriptional element. Nucleic Acids Res (2014) 42(16):10331–50. 10.1093/nar/gku696 25106871 PMC4176359

[119] Rasool R Ullah I Mubeen B Alshehri S Imam SS Ghoneim MM Theranostic interpolation of genomic instability in breast cancer. Int J Mol Sci (2022) 23(3):1861. 10.3390/ijms23031861 35163783 PMC8836911

[120] Rueda OM Sammut S-J Seoane JA Chin S-F Caswell-Jin JL Callari M Dynamics of breast-cancer relapse reveal late-recurring ER-positive genomic subgroups. Nature (2019) 567(7748):399–404. 10.1038/s41586-019-1007-8 30867590 PMC6647838

[121] Alba E Rueda OM Lluch A Albanell J Chin S-F Chacon JI Integrative cluster classification to predict pathological complete response to neoadjuvant chemotherapy in early breast cancer. American Society of Clinical Oncology (2018).

[122] van den Bosch T Rueda OM Caldas C Vermeulen L Miedema DM. Copy number heterogeneity identifies ER+ breast cancer patients that do not benefit from adjuvant endocrine therapy. Br J Cancer (2022) 127:1332–9. 10.1038/s41416-022-01906-3 35864159 PMC9519566

[123] Dugo E Piva F Giulietti M Giannella L Ciavattini A Gough L. Copy number variations in endometrial cancer: from biological significance to clinical utility. Int J Gynecol Cancer (2024) 34(7):1089–97. 10.1136/ijgc-2024-005295 38677776

[124] Heo Y Heo J Han SS Kim WJ Cheong HS Hong Y. Difference of copy number variation in blood of patients with lung cancer. Int J Biol Markers (2021) 36(1):3–9. 10.1177/1724600820980739 33307925

[125] Zhou C Zhang W Chen W Yin Y Atyah M Liu S Integrated analysis of copy number variations and gene expression profiling in hepatocellular carcinoma. Sci Rep (2017) 7(1):10570. 10.1038/s41598-017-11029-y 28874807 PMC5585301

[126] Li J Dittmar RL Xia S Zhang H Du M Huang CC Cell-free DNA copy number variations in plasma from colorectal cancer patients. Mol Oncol (2017) 11(8):1099–111. 10.1002/1878-0261.12077 28504856 PMC5537711

[127] Chen Y Sadasivan SM She R Datta I Taneja K Chitale D Breast and prostate cancers harbor common somatic copy number alterations that consistently differ by race and are associated with survival. BMC Med Genomics (2020) 13(1):116. 10.1186/s12920-020-00765-2 32819446 PMC7441621

[128] Hu W Li M Zhang Q Liu C Wang X Li J Establishment of a novel CNV-related prognostic signature predicting prognosis in patients with breast cancer. J Ovarian Res (2021) 14(1):103. 10.1186/s13048-021-00823-y 34364397 PMC8349487

[129] Borregales LD DeMeo G Gu X Cheng E Dudley V Schaeffer EM Response to takahashi. JNCI: J Natl Cancer Inst (2022) 114(11):1557–8. 10.1093/jnci/djac146 35880831 PMC9664183

[130] Yan K Niu L Wu B He C Deng L Chen C Copy number variants landscape of multiple cancers and clinical applications based on NGS gene panel. Ann Med (2023) 55(2):2280708. 10.1080/07853890.2023.2280708 37967237 PMC10653745

[131] Conrad B Antonarakis SE. Gene duplication: a drive for phenotypic diversity and cause of human disease. Annu Rev Genomics Hum Genet (2007) 8:17–35. 10.1146/annurev.genom.8.021307.110233 17386002

[132] Kallioniemi A Kallioniemi OP Sudar D Rutovitz D Gray JW Waldman F Comparative genomic hybridization for molecular cytogenetic analysis of solid tumors. Science (1992) 258(5083):818–21. 10.1126/science.1359641 1359641

[133] Eijk-Van Os PG Schouten JP. Multiplex Ligation-dependent Probe Amplification (MLPA®) for the detection of copy number variation in genomic sequences. Methods Mol Biol (2011) 688:97–126. 10.1007/978-1-60761-947-5_8 20938835

[134] Voutsadakis IA. Breast cancer sub-types display heterogeneity in gene amplification and mRNA expression of the anti-apoptotic members of BCL2 family. Gene (2023) 857:147179. 10.1016/j.gene.2023.147179 36627096

[135] Chen Y Olopade OI. MYC in breast tumor progression. Expert Rev Anticancer Ther (2008) 8(10):1689–98. 10.1586/14737140.8.10.1689 18925859 PMC3027840

[136] Reis-Filho JS Savage K Lambros MB James M Steele D Jones RL Cyclin D1 protein overexpression and CCND1 amplification in breast carcinomas: an immunohistochemical and chromogenic *in situ* hybridisation analysis. Mod Pathol (2006) 19(7):999–1009. 10.1038/modpathol.3800621 16648863

[137] Lamy PJ Fina F Bascoul-Mollevi C Laberenne AC Martin PM Ouafik L Quantification and clinical relevance of gene amplification at chromosome 17q12-q21 in human epidermal growth factor receptor 2-amplified breast cancers. Breast Cancer Res (2011) 13(1):R15. 10.1186/bcr2824 21288332 PMC3109584

[138] Rakha EA Armour JA Pinder SE Paish CE Ellis IO. High-resolution analysis of 16q22.1 in breast carcinoma using DNA amplifiable probes (multiplex amplifiable probe hybridization technique) and immunohistochemistry. Int J Cancer (2005) 114(5):720–9. 10.1002/ijc.20738 15609312

[139] Shahrouzi P Azimzade Y Brankiewicz-Kopcinska W Bhatia S Kunke D Richard D Loss of chromosome cytoband 13q14.2 orchestrates breast cancer pathogenesis and drug response. Breast Cancer Res (2024) 26(1):170. 10.1186/s13058-024-01924-4 39605038 PMC11600738

[140] Kang J. Genomic alterations on 8p21-p23 are the most frequent genetic events in stage I squamous cell carcinoma of the lung. Exp Ther Med (2015) 9(2):345–50. 10.3892/etm.2014.2123 25574196 PMC4280924

[141] Krig SR Miller JK Frietze S Beckett LA Neve RM Farnham PJ ZNF217, a candidate breast cancer oncogene amplified at 20q13, regulates expression of the ErbB3 receptor tyrosine kinase in breast cancer cells. Oncogene (2010) 29(40):5500–10. 10.1038/onc.2010.289 20661224 PMC4256946

[142] Pouladi N Abdolahi S Farajzadeh D Feizi MAHP. Association of the 17p13.1 region gene variants rs1042522 and rs2287499 with risk of breast cancer in Iranian-Azeri population. Meta Gene (2019) 19:117–22. 10.1016/j.mgene.2018.11.009

[143] Wang H Ba J Kang Y Gong Z Liang T Zhang Y Recent progress in CDK4/6 inhibitors and PROTACs. Molecules (2023) 28(24):8060. 10.3390/molecules28248060 38138549 PMC10745860

[144] Bruner HC Derksen PWB. Loss of E-cadherin-dependent cell-cell adhesion and the development and progression of cancer. Cold Spring Harb Perspect Biol (2018) 10(3):a029330. 10.1101/cshperspect.a029330 28507022 PMC5830899

[145] S Zeind C. Applied therapeutics for clinical pharmacists. Wolters Kluwer (2018).

